# Acute telomere deprotection prevents ongoing BFB cycles and rampant instability in p16^INK4a^-deficient epithelial cells

**DOI:** 10.18632/oncotarget.25502

**Published:** 2018-06-05

**Authors:** Aina Bernal, Marc Moltó-Abad, Daniel Domínguez, Laura Tusell

**Affiliations:** ^1^ Unitat de Biologia Cel·lular, Facultat de Biociències, Universitat Autònoma de Barcelona, 08193 Cerdanyola del Vallès, Spain; ^2^ Current address: Unitat de Malalties Minoritàries, Hospital Universitari de la Vall d’Hebron, 08035 Barcelona, Spain

**Keywords:** MCF-10A, breast epithelial cells, chromosome instability, telomere-dysfunction, TRF2^ΔBΔM^

## Abstract

Telomere dysfunction drives chromosome instability through endless breakage-fusion-bridge (BFB) cycles that promote the formation of highly rearranged genomes. However, reactivation of telomerase or ALT-pathway is required for genome stabilisation and full malignant transformation. To allow the unrestricted proliferation of cells at risk of transformation, we have established a conditional system of telomere deprotection in p16^INK4a^-deficient MCF-10A cells with modified checkpoints. After sustained expression of a dominant negative form of the shelterin protein TRF2 (TRF2^ΔBΔM^), cells with telomere fusion did progress to anaphase but no signs of ongoing BFB cycles were observed, thus anticipating proliferation defects. Indeed, 96 h TRF2^ΔBΔM^ expression resulted in noticeable growth proliferation defects in the absence of cell cycle disturbances. Further transient periods of 96 h telomere uncapping did not result in cell cycle disturbances either. And reduction of the telomere damage to short acute deprotection periods did not in any case engender cells with a reorganised karyotype. Strikingly, the growth arrest imposed in cells showing dysfunctional telomeres was not accompanied by an activation of the DNA damage response at cellular level, or by the presence of visible markers of senescence or apoptosis. We propose that the deprotection of many telomeres simultaneously, even for a short time, results in a local activation of the cellular stress response which consequently triggers gradual cell withdrawal from cell cycle, restraining the onset of genomic instability.

## INTRODUCTION

Telomeres are nucleoprotein complexes that cap the ends of chromosomes, thus preventing illegitimate recombination processes. By adopting a t-loop structure, telomeres ensure genomic stability by providing chromosome end protection [1, 2, and reviewed by 3]. The most deleterious outcome of telomere deprotection is the formation of chromosome end-to-end fusions, which may fire breakage-fusion-bridge (BFB) cycles and rampant chromosome instability (CIN) [4–6, and reviewed by 7]. In certain types of epithelial cancers, telomere dysfunction is considered to be a key trigger for CIN and a promoter of tumourigenesis [8, 9]. Specifically in the breast, studies support the view that telomere dysfunction can precede disease progression and is not simply a biomarker of advanced disease [10–13]. Indeed, qFISH studies have indicated modest telomere shortening occurring in hyperplasia, a more significant reduction becoming prevalent as early as ductal carcinoma *in situ* (DCIS) [14, 15], and the presence of significantly short telomeres in malignant breast cells compared to normal surrounding breast tissue [16]. The impact of telomeres in breast carcinogenesis is further supported by the detection of telomere-to-telomere fusion, a hallmark of telomere dysfunction, in early stage breast tumours, including DCIS [17].

Telomeres that can no longer exert end-protective functions because of excessive telomere attrition or alterations in the components of the shelterin complex itself, are recognised as sites of DNA damage and recruit the same repair factors that are associated with double strand breaks (DSBs) at other sites of the genome [18, 19]. Unprotected chromosome ends impinge on signalling kinases ATM and ATR to activate a DNA damage response (DDR) that via p53-p21^Waf1/Cip1^ or pRb-p16^INK4a^ axis leads to checkpoint-mediated cell cycle arrest and senescence or apoptosis [20, 21]. Among the shelterin proteins, TRF2 (telomere repeat binding factor 2) is at the heart of the molecular events that maintain telomere integrity in mammals [22–24, and reviewed by 25]. TRF2 binding to DNA *in vitro* stimulates strand invasion, adopting structures that resemble t-loops [2]. Furthermore, the frequency of t-loops *in vivo* is significantly reduced in cells lacking TRF2, implicating this sheltering subunit in its formation and/or stabilisation [26]. It has been previously reported that expression of the truncated form of TRF2 (TRF2^ΔBΔM^), which lacks the Basic and Myb domains, interferes with the accumulation of the endogenous TRF2 protein at telomeres [22]. Depletion of TRF2 in normal cells using RNAi, dominant-negative alleles or Cre-mediated deletion typically results in a non-reversible telomere dysfunction phenotype that induces strong DNA damage signalling and stalls cell cycle progression [19, 22, 23, 27]. Therefore, telomere dysfunction acts as a tumour suppressive mechanism in cells with a functional DDR by limiting the expansion of unstable cell populations harbouring precancerous mutations. In sharp contrast, dysfunctional telomeres in cells with a limited DDR might allow the proliferation of damaged cells at risk of transformation if telomere length is stabilised through telomerase activation or ALT-pathways.

With the aim of generating heavily rearranged but telomerase stabilised epithelial human cells, we generated a versatile experimental system of telomere deprotection where TRF2^ΔBΔM^ expression is controlled by a doxycycline inducible promoter in the non-tumorigenic epithelial mammary cell line MCF-10A. We reasoned that limiting the telomere insult to brief periods might allow for a bypass of the acute cellular responses to dysfunctional telomeres. Besides that, given that telomere dysfunction can either prevent or promote tumourigenesis depending on the intactness of the DDR system, we used different approaches to experimentally inhibit the p53/pRb pathways. Our results demonstrate that, after 96 h of sustained TRF2^ΔBΔM^ expression, the telomere dysfunction phenotype increased with checkpoint protein inactivation, with the greatest impact seen in SV40LT transduced MCF-10A cells. However, evidence of chromosome specific structural aberrations or extensive aneuploid configurations compatible with ongoing BFB cycles were unnoticed in cells lacking p16^INK4a^ only or along with p53 inactivation, thus supporting the incapacity of p16^INK4a^-deficient cells to cope with acute telomere damage. Even periods of short acute telomere deprotection did not dramatically alter the cell cycle profile of p16^INK4a^-deficient cells or give rise to an intensification of the telomere-dependent CIN over time. Collectively, this indicates that cells experiencing transient acute telomere damage cannot overcome the severe proliferation defect imposed by uncapped telomeres and are destined to die.

## RESULTS

The MCF-10A cell line is a spontaneously immortalised, but non-transformed human mammary epithelial cell line derived from breast tissue [28]. This cell line maintains telomere length through telomerase, but its expression is low [29, 30], making it hard to see a clear band of hTERT by western blotting (Supplementary Figure 1). Furthermore, despite being commonly recognised as normal cells, the karyotype is cytogenetically abnormal (Supplementary Figure 2A) and harbours genetic abnormalities commonly associated with cultured mammary epithelial cells such as deletion of the locus containing p16^INK4a^ and p14^ARF^, as well as MYC amplification [31, 32].

### Establishment of conditional TRF2^ΔBΔM^ MCF-10A cell lines with different cell cycle settings

**Figure 1:**
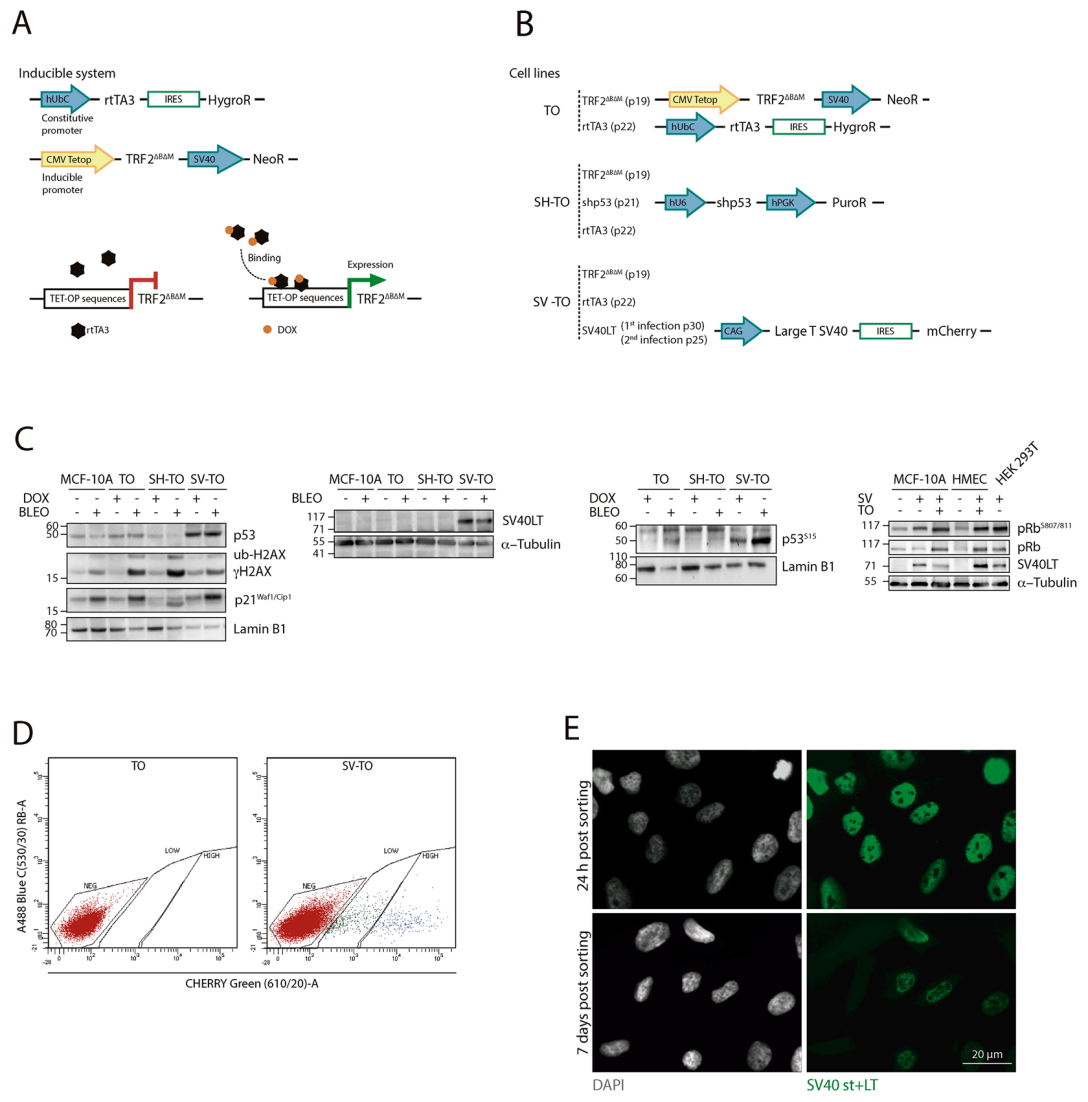
Generation of different MCF-10A cell lines with inducible TRF2^ΔBΔM^ expression **(A)** Scheme of the conditional TET-ON expression system for TRF2^ΔBΔM^. The doxycycline-inducible system consisted in the constitutive expression of the rtTA3 protein through the hUbC (Ubiquitin C) promoter and the TRF2^ΔBΔM^ protein under the inducible CMV (Citomegalovirus)-Tetop promoter. In the absence of the tetracycline-derivative doxycycline (DOX), the rtTA3 protein is unable to bind to the TET-OP sequences located at the promoter; consequently, gene expression is repressed. In contrast, when DOX is added to the cell culture, it binds to rtTA3, which subsequently undergoes a conformational change that allows it to bind to the inducible promoter, starting TRF2^ΔBΔM^ transcription. **(B)** All cell lines (TO; SH-TO and SV-TO) contained the doxycycline-expression system. In addition to these modifications, SH-TO cell lines were transduced with the short hairpin RNA of p53 under the hU6 constitutive promoter. And the SV-TO cell line expressed the SV40 Large T antigen and mCherry under the CAG (CMV enhancer-chicken beta actin) constitutive promoter. All constructs contained a selectable marker or a resistence gene for selection purposes, under the same or a different promoter. The inducible MCF-10A cell lines (TO; SH-TO; SV-TO) were generated through serial transduction with TRF2^ΔBΔM^ (passage 19), rtTA3 (passage 22), shp53 (passage 21) or SVLT40-mCherry (passage 25 and 30) lentivirus. **(C)** Immunoblots of untreated and DOX- or Bleocin™-treated MCF-10A and TO, SH-TO and SV-TO cell lines. Diminished levels of p53 were observed in the SH-TO cell line as well as reduced levels of p53^S15^ and p21^Waf1/Cip1^ after DSBs induction by Bleocin™, thus validating short hairpin RNA p53 inactivation. In SV-TO cells, western blots confirmed the presence of SV40 Large T antigen. In this cell line, higher p53 and p53^S15^ levels are observed due to p53 stabilisation by LT antigen. After DSBs induction by Bleocin™ exposure, both TO and SV-TO cells showed enhanced p53^S15^ and p21^Waf1/Cip1^ levels. The loss of the G1 growth suppressor function of pRb was determined by the ratio between pRb^S807-811^/total pRb. Cell lines containing the SV40LT antigen showed higher pRb^S807/811^ levels than uninfected cell lines, thus supporting inactivation of pRb protein. **(D)** SV-TO cells were selected by FACS of cells expressing mCherry. SV40LT-mCherry uninfected TO cells were used as control to discard cell autofluorescence. The FACS profile of SV-TO MCF-10A cell line shows weakly and strongly mCherry expressing populations. For the following experiments, SV-TO cells with strong mCherry expression were used. **(E)** SV40 T-antigens (st+LT) immunofluorescence in SV-TO cells 24 h and one week after FACS sorting. Immunofluorescent images, captured under the same exposure conditions, denote the partial loss of T-antigen expression over time. Scale bar corresponds to 20 μm.

Different MCF-10A cell lines were generated to display telomere dysfunction in a regulated manner through doxycycline (DOX)-induced expression of TRF2^ΔBΔM^ (Figure 1A). The MCF-10A T/O TRF2^ΔBΔM^ (TO) cell line was generated after serially transducing MCF-10A cells with lentiviral particles containing the inducible TRF2^ΔBΔM^ cassette and the rtTA3 transactivator (Figure 1B). Because abrogation of the p53 and pRb pathways is needed in human cells to bypass senescence and to initiate rampant telomere-dependent CIN, we established a second MCF-10A T/O TRF2^ΔBΔM^ inducible cell line where p53 expression was constitutively abolished using short-hairpin p53 RNA lentiviral particles (SH-TO) (Figure 1B). After antibiotic selection, diminished levels of p53 were verified through western blotting (Figure 1C and Supplementary Figure 3A). Besides that, inactivation of the p53 pathway was confirmed by the fact that increased levels of p53^S15^ and p21^Waf1/Cip1^ were practically unnoticed after cell exposure to the DSBs inducer Bleocin™ (Figure 1C and Supplementary Figure 3A) and by the increased ability of tetraploid cells to re-enter the cell cycle and initiate another round of DNA replication after 24 h colcemid exposure and release (Supplementary Figure 4). Moreover, as an independent way of inactivating the pRb and p53 pathways, we generated a third TRF2^ΔBΔM^ conditional MCF-10A model (SV-TO) by transducing TO cells with a lentivirus that constitutively expresses the Large T antigen gene from SV40 and mCherry (Figure 1B). The translation product of SV40LT functions as a viral oncoprotein, which upon binding to p53 and pRb proteins inhibits their functions [33]. Selection of SV40LT containing cells was done by fluorescence activated cell sorting (FACS) of mCherry positive cells (Figure 1D) and afterwards SV40LT expression was validated by western blot, immunofluorescence and flow cytometry. As expected, the Large T antigen was present in SV-TO whole cell extracts and detected in the nucleus of transduced cells by immunofluorescence (Figure 1C and 1E). However, we found that SV40LT expressing cells were gradually lost from the culture (Figure 1E). Additional experiments with the p16^INK4a^-deficient MCF-10A cells demonstrated again the loss of SV40LT positive cells with time (Supplementary Figure 5A and 5B). This leaking effect was previously reported to occur in p16^INK4a^-deficient human mammary epithelial cells (vHMECs) transduced with SV40 early region (st and LT antigens) [34, 35]. To validate this, HMECs derived from cosmetic breast reductions were transduced with the same SV40LT antigen-mCherry vector. In this case, HMECs-hTERT were infected at an early population doubling (PD 6.92) before p16^INK4a^ inactivation takes place and we did not pick clones or select for mCherry cells by FACS. In those HMECs-hTERT-SV40LT, the leaking effect was not observed. But most importantly, the analysis of SV40LT antigen levels by western blotting and flow cytometry at PD>92.31, demonstrated that cells were still positive for the Large T antigen (Supplementary Figure 5B and 5C). As a whole, and confirming previous studies, breast epithelial cells p16^INK4a^-deficient are refractory to SV40 transformation. The reason why this occurs is unknown and further experiments would be needed to ascertain it.

Even though the generation of a stable SV-TO cell line over time was unsuccessful, at the time of the analysis, immunoblots revealed the presence of the SV40LT antigen (Figure 1C). The infection of cells with Large T antigen presumably renders the cells oblivious to the DNA damage checkpoint by inactivating both pRb and p53. SV40LT antigen triggers p53^S15^ and stabilises p53 protein, but it is suggested that the direct interaction between SV40LT and p53 inhibits its function as a transcription factor [36]. In SV-TO cells, immunoblots demonstrated stabilisation of p53 by a higher expression of both p53^S15^ and p53 in comparison with the other cell lines (Figure 1C and Supplementary Figure 3A). However, after Bleocin™ treatment the cells were capable of upregulating p53^S15^ and p21^Waf1/Cip1^ (Figure 1C and Supplementary Figure 3A). Given that it was not clear whether upregulation was due to the improper p53 pathway inactivation or to the presence of SV-TO cells lacking the SV40LT antigen, the response of SV-TO cells to acute colcemid treatment was also evaluated. Coincident with SH-TO cells, a significant polyploid population was observed in SV-TO cells 48 h after colcemid treatment (Supplementary Figure 4) thus supporting that at least the p53 pathway was abrogated in some cells. To determine the functional inactivation of the pRb pathway we tested the expression of the retinoblastoma pocket protein pRb (p105) and its phosphorylated form pRb^S807/811^ (Figure 1C), as Large T antigen binds and inactivates pRb pocket proteins [37]. The ratio of pRb^S807/811^/pRb was low in those cells not containing the SV40LT antigen (Supplementary Figure 3B). In cells expressing the LT antigen, pRb was in a more hyper-phosphorylated state and higher pRb^S807/811^/pRb ratios were detected, thus suggesting alleviation of the pRB-mediated repression checkpoint.

Finally, the specific genetic changes present in the parental MCF-10A cell line were evaluated (Supplementary Figure 2A). The modal karyotype at p15 was defined as 47, XX, i(1q), del(1q), +1, der(3)t(3;9), der(8)t(8;8), der(9)t(9;3;5). In addition to these clonal aberrations, previously described in the literature [38–40], some signs of CIN were found. A total of 10.81% of non-modified MCF-10A metaphases showed non-clonal chromosome aberrations including chromosome fragments, chromatid breaks and one dicentric chromosome that showed telomeric FISH signals at the fusion point (Supplementary Table 1). Karyotyping by reverse DAPI banding was also performed in the uninduced TO, SH-TO and SV-TO cell lines to determine the cytogenetic impact of the genetic modifications (Supplementary Figure 2 and Supplementary Table 1). Collectively, these data suggested that the establishment of the TET-ON inducible system did not have a deleterious effect on the karyotype of MCF-10A epithelial cells. By contrast, and as previously reported [41], diminished levels of p53 resulted in an increase of cells containing chromosome aberrations with regard to the parental MCF-10A (Supplementary Figure 2). This adverse effect was markedly opposed if disruption was achieved through short hairpin RNA interference or SV40LT infection. Whereas shp53 resulted in an increase in non-clonal unstable aberrations that included rejoined broken chromosomes, acentric fragments and chromosome breaks, SV40LT transduction resulted in clonal stable chromosome aberrations (Supplementary Figure 2). This divergent result could be explained by the time elapsed between infection and the cytogenetic analysis. Due to the loss of SV40LT expression, SV-TO cells were analysed shortly after transduction and sorting, while much time passed between infection, selection and chromosome analysis in the SH-TO cell line. Given that the chromosome damage induced by SV40LT is an active process that gradually increases with serial cell passage [42] (Supplementary Figure 5D), the prompt analysis of the SV-TO cell line after transduction avoided the adverse evolution of the karyotype.

### Variable intensity of telomere dysfunction upon 96 h sustained TRF2^ΔBΔM^ expression in the modified MCF-10A cell lines

**Figure 2:**
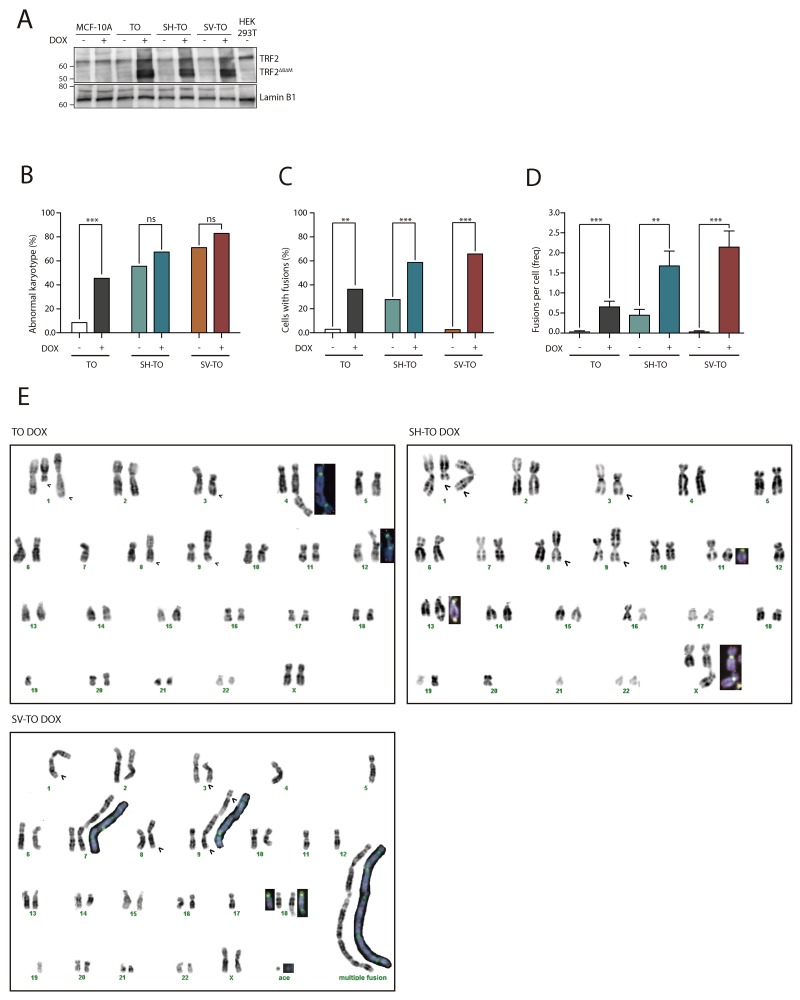
TRF2^ΔBΔM^ expression induces chromosome end-to-end fusions in all inducible cell lines **(A)** Immunoblots of MCF-10A, TO, SH-TO and SV-TO cell lines with and without DOX and HEK 293T. After 96 h of DOX treatment, the inducible cell lines expressed the truncated TRF2^ΔBΔM^ protein (50 kDa); in contrast, uninduced cell lines, MCF-10A parental cell line and HEK 293T cells displayed only the endogen TRF2 protein (66 kDa). Lamin B1 was used as loading control. **(B)** After sustained expression of TRF2^ΔBΔM^ for 96 h there was a significant increase in aberrant metaphases only in TO cells when compared to uninduced matched cells. **(C)** Nevertheless, the efficacy of the inducible system was validated by the statistical increase of cells with end-to-end fusions in all inducible cell lines. **(D)** Moreover, a high incidence of fusions per cell was found in all modified cell lines after sustained TRF2 depletion. Data was presented as mean + SEM. **(E)** Example of TO, SH-TO and SV-TO karyotypes after 96 h of TRF2^ΔBΔM^ expression. Open arrows indicate clonal aberrations in the parental MCF-10A cell line. Insets in the karyotype show rearranged chromosomes stained with centromeric (green) and telomeric (red) PNA probes. Note the presence of telomere FISH signals at the fusion point of chromatid- or chromosome-type end-to-end fusions.

To validate the efficacy of the inducible system, we exposed the parental MCF-10A and the three modified cell lines to doxycycline for 96 h. The expression of the truncated TRF2^ΔBΔM^ protein upon DOX addition was demonstrated by immunoblotting protein extracts from untreated and DOX-treated cells with full length TRF2 antibodies (Figure 2A and Supplementary Figure 3C).

**Figure 3:**
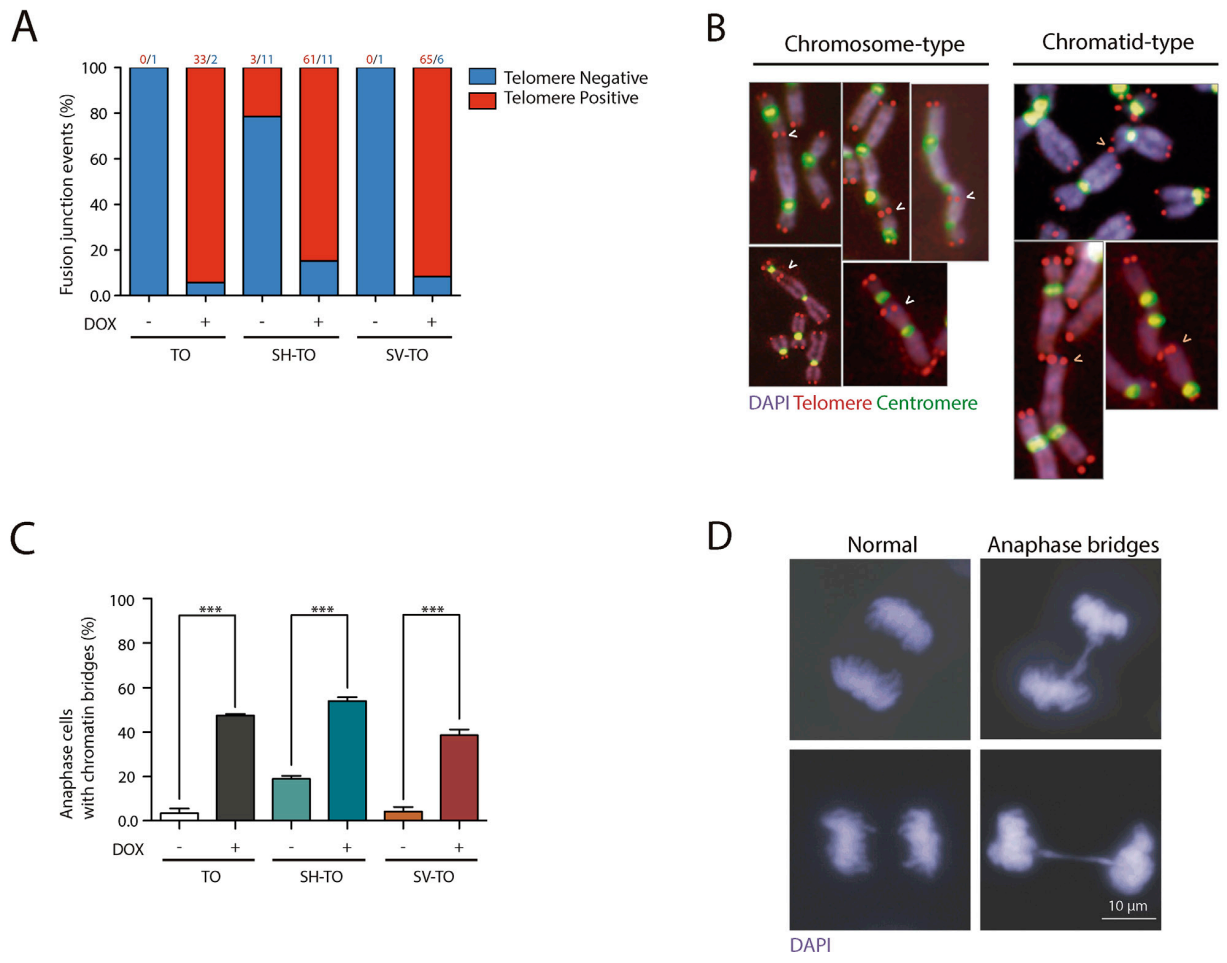
TTAGGG-positive fusions and anaphase bridges after sustained TRF2^ΔBΔM^ expression **(A)** TRF2^ΔBΔM^ expression significantly increased the percentage of fusion events displaying TTAGGG repeats at the fusion point, thus confirming that fusions occurred because of the unfolded t-loop structure and not telomere DNA shortening. The number of events of each category is shown above the bars. **(B)** TRF2 stripped telomeres mostly fused at the G1 cell cycle phase, giving rise to chromosome-type fusions. Chromatid-type fusions that formed in G2 were also observed, although to a lower extent. **(C)** The expression of TRF2^ΔBΔM^ also increased the proportion of anaphase cells with chromatin bridges, another marker of telomere dysfunction. Data are presented as mean + SEM from three independent experiments. **(D)** Representative images of anaphase cells without (left) and with (right) chromatin bridges. Scale bar corresponds to 10 μm.

Then, the telomere dysfunction phenotype was evaluated through a deep cytogenetic analysis of metaphase spreads from 96 h DOX-treated and uninduced matched cells. Overall, the frequency of aberrant metaphases significantly increased in the TO cell line expressing TRF2^ΔBΔM^ (8.57% vs. 45.45%; p= 0.0002), but strikingly this was not the case for SH-TO (55.56% vs. 67.39%; p= 0.3594) nor for SV-TO (71.05% vs. 82.86%; p= 0.2767) (Figure 2B and Supplementary Table 1). This observation was probably due to the high frequency of abnormal karyotypes induced by p53 or p53/pRb inactivation. Indeed, clear evidence of the telomere dysfunction phenotype was observed when only metaphase spreads showing chromosome fusions were considered (Figure 2C). TRF2^ΔBΔM^ expression for 4 days induced a significant increase in metaphase cells showing end-to-end fusions (TO p= 0.0002; SH-TO p= 0.0072 and SV-TO p< 0.0001) (Supplementary Table 1). On average, there were 0.65 fusion events per cell in TO, 1.67 in SH-TO and 2.14 in SV-TO after expression of the dominant negative form of TRF2, which is statistically higher than the rates observed in matched uninduced cell lines (Mann-Whitney U-test; TO p= 0.0003; SH-TO p= 0.0035 and SV-TO p< 0.0001) (Figure 2D and Supplementary Table 1). Chromosome-type fusions, i.e. dicentric chromosomes formed during G1 were more frequently observed than chromatid-type fusions, which typically originate in the G2 phase of the cell cycle (Supplementary Figure 6). Usually both types of fusions involved two chromosomes, though multiple concatenated chromosomes were occasionally observed in the SH-TO and SV-TO cell lines (Figure 2E). At that point, in order to unambiguously demonstrate that telomere fusions were due to chromosome ends lacking sufficient TRF2 protection, we performed PNA-FISH experiments with pancentromeric and pantelomeric PNA-probes. In uninduced cells, most fusion events did not display TTAGGG FISH signals at the junction point, probably indicating DSB-DSB rejoining as origin. By contrast, after TRF2^ΔBΔM^ expression, the vast majority of fusion events displayed telomeric FISH signals at the fusion point (94.29% telomere positive fusions in TO, 84.72% in SH-TO and 91.55% in SV-TO) (Figure 3A and 3B, and Supplementary Table 2), thus demonstrating the presence of telomeric DNA and validating the efficacy of our telomere dysfunction inducible system.

Additionally, the telomere dysfunction phenotype was assessed by analysing the presence of anaphase bridges. A minimum of 215 anaphases were examined in uninduced and doxycycline-treated modified cell lines (Supplementary Table 3). The presence of cells with chromatin bridges during anaphase was low in TO and SV-TO cells not expressing the truncated TRF2 protein (3.72% and 4.21%, respectively). By contrast, the percentage of cells displaying anaphase bridges in uninduced SH-TO increased to 18.93%, which is statistically higher than the others (p< 0.0001) (Figure 3C and 3D, and Supplementary Table 3). This agrees with the high frequency of chromosome fusion events displayed by SH-TO cells when TRF2^ΔBΔM^ was not induced (Supplementary Table 1). After 96 h of acute telomere dysfunction, there was a significant increase in cells containing anaphase bridges in all the cell lines expressing TRF2^ΔBΔM^ when compared with matched uninduced cell lines (p< 0.0001) (Figure 3C and 3D, and Supplementary Table 3).

Overall, the expression of TRF2^ΔBΔM^ during 96 h leads to a telomere-dysfunction phenotype whose level of intensity depends on the functionality of cell cycle checkpoints. TO cells, which are p16^INK4a^-deficient but present wild-type p53, showed the lowest percentage of cells with end-to-end fusions, as well as the smallest frequency of fusions per cell. In sharp contrast, the telomere-dysfunction phenotype was exacerbated in the SV40 LT antigen transduced cells, thus confirming that disabled cell-cycle checkpoints allow the accumulation and survival of cells containing telomere damage.

### Acute telomere deprotection fires BFB cycles but prevents the development of CIN

Cells undergoing telomere dysfunction set in motion BFB cycles that, following chromatin bridge resolution, give rise to chromosome structural aberrations, gains and losses of chromosomes (aneuploidy) and regional amplification (reviewed by [7, 43]). Most recently, chromothripsis and kataegis have also been documented to occur after chromatin bridge fragmentation [44]. Besides that, several studies in human cells and in the mouse have clearly shown that deprotection of chromosome ends leads to polyploidisation events [45, 46].

To evaluate the presence of telomere-dependent CIN we analysed for BFB cycles scars in the form of structural chromosome aberrations other than end-to-end fusions. Structural reorganisations such as non-clonal non-reciprocal translocations (NRT) were few and did not increase after TRF2^ΔBΔM^ expression. This result was quite unexpected, given the significant increase of cells with chromatin bridges after telomere damage.

**Figure 4:**
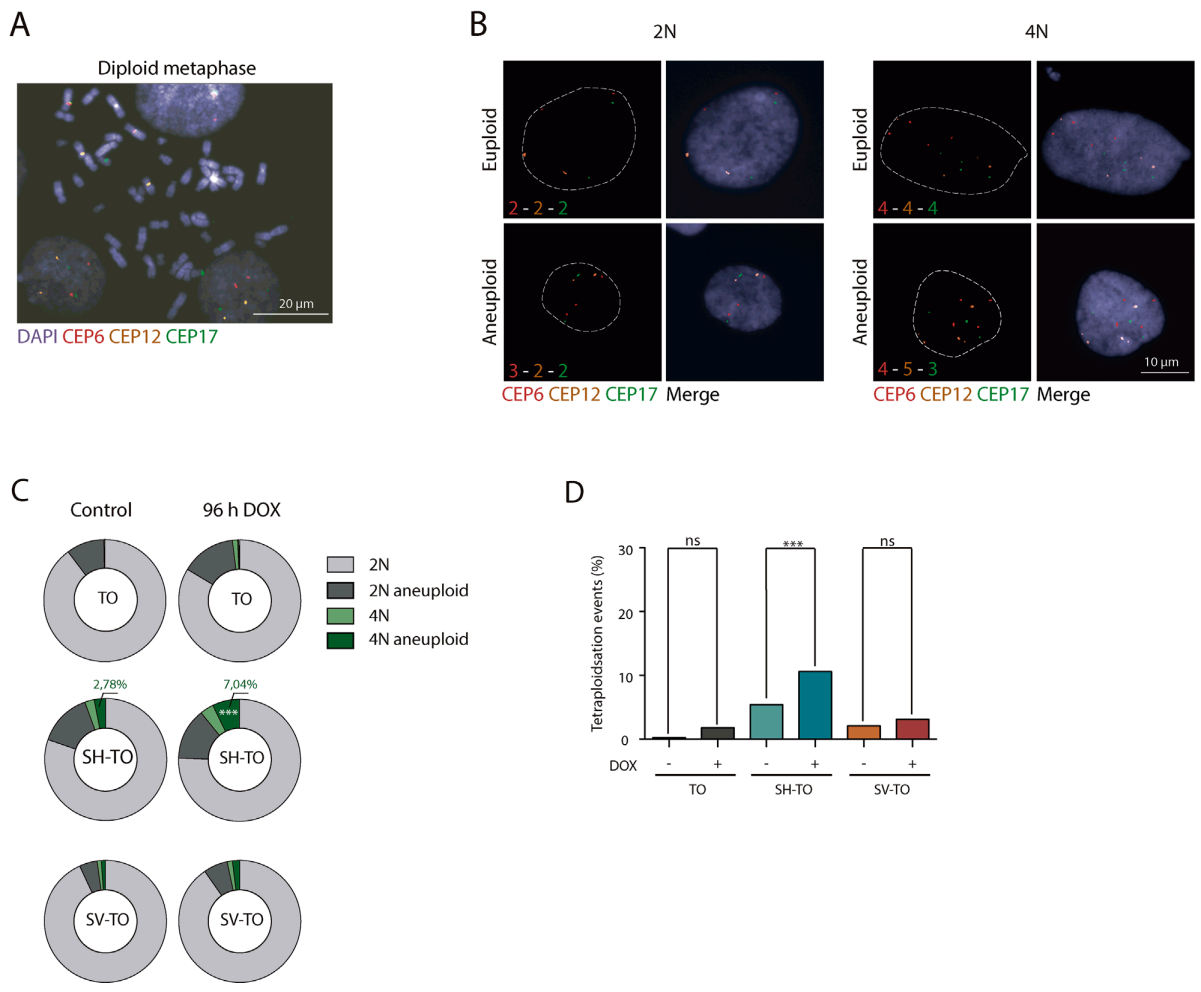
Tetraploidisation is not the usual fate of cells experiencing sustained TRF2^ΔBΔM^ expression **(A)** Representative image of a diploid metaphase, showing the specificity of the centromeric probes tested: chromosomes 6 (red), 12 (yellow) and 17 (green). Scale bar corresponds to 20 μm. **(B)** Representative images of diploid and tetraploid cells with euploid and aneuploid configurations of tested centromeric probes. Scale bar corresponds to 10 μm. **(C)** Aneuploid configurations after TRF2 depletion only increased in the 4N fraction of SH-TO cells. **(D)** Percentage of tetraploid cells before and after TRF2^ΔBΔM^ expression. Tetraploid cells significantly increased in the SH-TO cell line after DOX treatment. In contrast, this was not the case for TO or SV-TO cell lines (p= 0.0375 and p= 0.3122, respectively; Bonferroni p-value correction= 0.0167).

Besides that, FISH analysis with three different centromeric specific probes was also conducted to ascertain whether TRF2^ΔBΔM^ expression engendered numerical chromosome changes. Chromosomes #6, #12 and #17 were selected for their uninvolvement in numerical aberrations in the parental MCF-10A and uninduced cell lines (Supplementary Figure 2). The specificity of the centromeric probes was determined on metaphase chromosomes (Figure 4A) and aneuploid configurations were evaluated in a minimum of 284 interphase nuclei of treated and untreated cells (Supplementary Table 4). The percentage of 2N aneuploid cells ranged from 4.66% to 14.10% and, upon doxycycline addition, this fraction did not increase in any of the modified cell lines (p= 0.0743, p= 0.9162 and p= 0.2567, for TO and SH-TO and SV-TO, respectively) (Figure 4B and 4C). Tetraploid events in uninduced TO cells were few (Figure 4C and 4D), but according to the role of p53 in limiting the proliferation of polyploids, the fraction of tetraploid cells in untreated SH-TO and SV-TO cells was significantly higher than in TO (p< 0.0001 and p= 0.0094, respectively). Nevertheless, tetraploidisation events in SV-TO cells were significantly fewer than those in SH-TO cells, which was unexpected considering that SV40LT also inhibits p53 function. Strikingly, upon TRF2^ΔBΔM^ expression the fraction of tetraploid cells was exacerbated only in SH-TO cells (p= 0.0099). This result was quite surprising as given the elevated level of end-to-end fusions in SV-TO metaphases, we rather expected similar results for the SV-TO cell line. However, such was not the case, and indeed we found that the fraction of 4N cells in induced SV-TO was similar to that observed in treated TO cells (p= 0.3349). Moreover, the fraction of tetraploid cells displaying aneuploid configurations increased upon TRF2^ΔBΔM^ expression when p53 was attenuated (2.78% vs. 7.04% in SH-TO and 0.86% vs. 1.91% in SV-TO), however statistical significance was only denoted for SH-TO cells (p= 0.0059) (Figure 4C).

As a whole, chromosome banding of metaphase spreads and centromeric-specific FISH analysis in interphase nuclei demonstrated the failure of long-term telomere deprotection to induce extensive structural and numerical changes compatible with ongoing BFB cycles. Therefore, these data suggest that sustained 96 h of acute telomere deprotection prevents active proliferation of cells suffering telomere damage.

### Transient cycles of 96 h acute telomere damage results in proliferation defects in the absence of cell cycle alterations, DDR activation and visible senescent and apoptotic markers

Whereas persistent disruption of TRF2 activates a DDR that signals cell cycle arrest or apoptosis [23, 24], the fate of cells experiencing transient periods of acute telomere deprotection remains unclear. To this end, cell cycle progression studies were conducted in the modified MCF-10A cell lines after switching on/off TRF2^ΔBΔM^ expression. First, we analysed DOX-mediated cell cycle profile changes in unmodified MCF-10A cells to assess potential detrimental effects of DOX on cell proliferation [47]. After 24 h or 96 h exposure, we did not find evidence that 1 μg/ml DOX affected the cell cycle profile of unmodified MCF-10A cells (Mann-Whitney U-test; p> 0.05) (Supplementary Figure 7).

**Figure 5:**
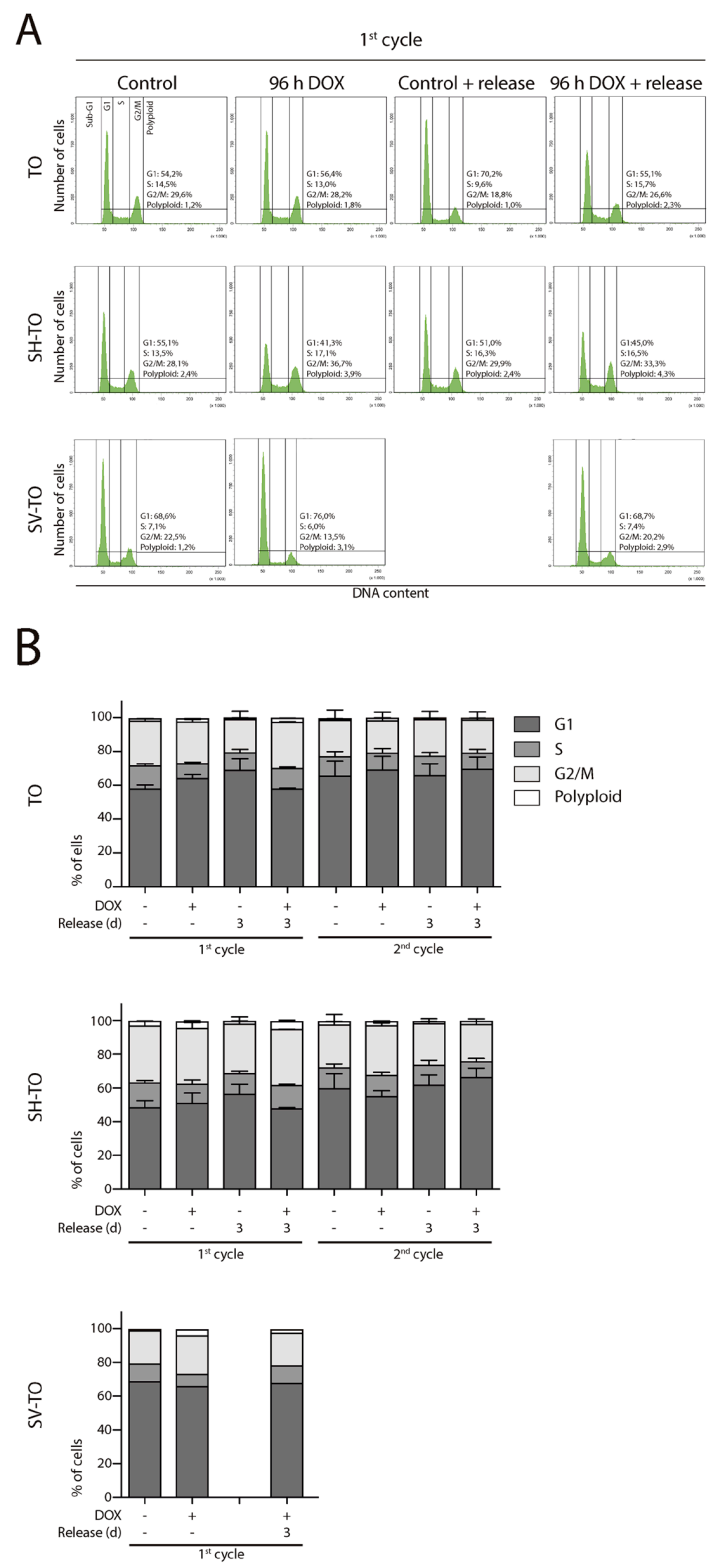
Absence of cell cycle profile disturbances after long TRF2^ΔBΔM^ expression periods **(A)** Representative cell cycle profiles of TO, SH-TO and SV-TO cell lines during the first cycle of 96 h DOX treatment and their respective controls. Cell cycle profiles remained stable throughout the time of the experiment. Cell cycle phases are marked and values indicated. **(B)** Cell cycle phases distribution among TO, SH-TO cell lines (first and second cycle) and SV-TO cell line (first cycle) after 96 h DOX experiments. No statistical differences were observed between control and treated samples (Mann-Whitney test). A minimum of 10,000 cells were analysed per experiment. Data are presented as mean + SEM from three independent experiments, except for SV-TO cell line, in which only one experiment is shown.

**Figure 6:**
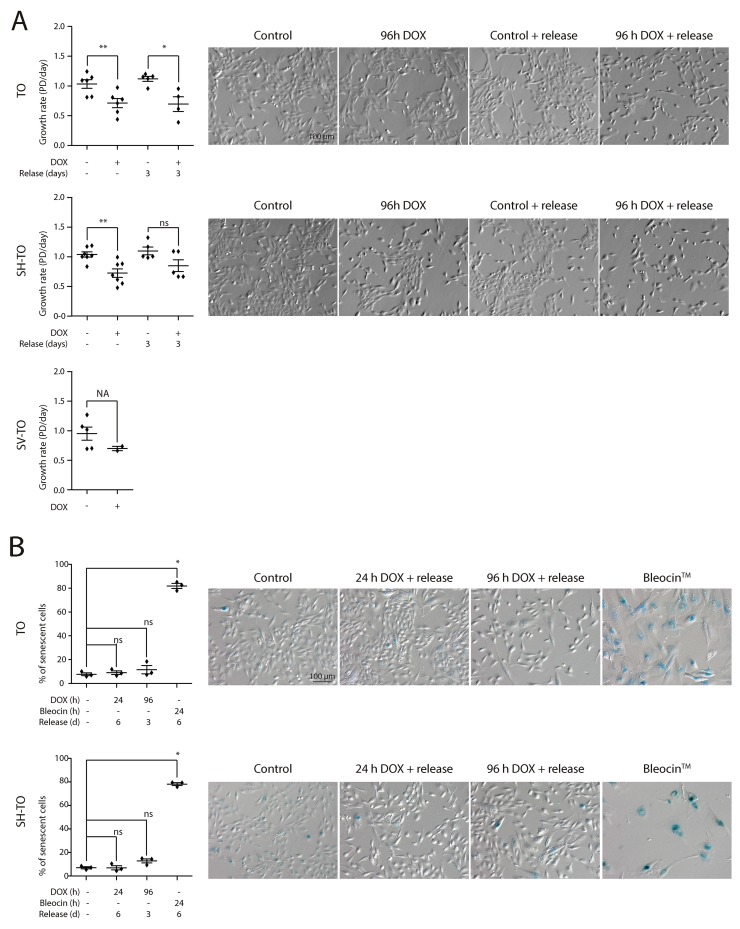
Reduced growth of cells displaying acute telomere dysfunction in the absence of a senescent phenotype **(A)** Growth rate experiments in the modified cell lines after 96 h DOX exposure or after three days release from exposure demonstrate a reduced proliferation of cells displaying uncapped telomeres (TO cell line: 96 h DOX p= 0.0087, 96 h DOX and release p= 0.0159; SH-TO cell line: 96 h DOX p= 0.0032, 96 h DOX and release p= 0.2222). Analysis was performed from at least three independent experiments and analysed by Mann-Whitney test. Statistical analysis of SV-TO cells was not assessable (NA) due to the reduced number of replicates. At the right side, representative contrast field images of control and treated cells. Scale bar corresponds to 100 μm. **(B)** SA-β-galactosidase activity staining was analysed only in TO and SH-TO cell lines. The cells were exposed to 24 h or 96 h DOX and analysed after six or three day release, respectively. As positive control, the cells were treated with the DSBs-inducer Bleocin™ during 24 h and processed 6 days later. Analysis was performed on three replicates and Kuskal-Wallis test and Dunn’s multiple comparison post-test. At the right side, representative bright field images of control and treated cells. Scale bar corresponds to 100 μm.

Then, asynchronous untreated cultures, subjected to doxycycline for 96 h, as well as those recovered after one or two cycles of 96 h DOX exposure and washout, were monitored by FACS analyses of DNA content. No significant alterations were observed in the cell cycle profile of the modified cell lines after 96 h of sustained TRF2^ΔBΔM^ expression or after three days release (first DOX cycle) (Mann-Whitney U-test; p> 0.05) (Figure 5A and 5B). Moreover, exposure of TO and SH-TO cells to a second cycle of TRF2^ΔBΔM^ expression did not perturb the cell cycle profile either (Mann-Whitney U-test; p> 0.05). These observations agreed with the absence of changes in the S-phase index after 96 h telomere damage analysed through incorporation of BrdU (data not shown, only one experiment). Although the cell cycle arrest was not detected, TRF2^ΔBΔM^ sustained expression during four days resulted in a significant reduction of cell proliferation in both TO and SH-TO cells (Mann-Whitney U-test; TO p= 0.0087; SH-TO p= 0.0032) (Figure 6A). Despite the fact that end-to-end telomere fusions can slow progression through mitosis thus misleading cell proliferation changes, the absence of genomic instability compatible with ongoing BFB cycles also supports the notion that cells stop proliferation and ultimately senesce or die. However, no obvious senescent morphology was observed in the modified MCF-10A cells undergoing TRF2 depletion (Figure 6A). Furthermore, TRF2^ΔBΔM^ expressing cells were indistinguishable from control cultures by SA-β-galactosidase activity staining (Kruskal Wallis and Dunn’s multiple comparison test; p> 0.05) (Figure 6B), which agrees with studies reporting abrogation of senescence in p16^INK4a^-deficient cells [48]. These results together with the observation that apoptosis is triggered in p16^INK4a^-deficient mammary adenocarcinoma MCF7 cell line upon TRF2^ΔBΔM^ expression [23], evoked that the reduced proliferation in the modified MCF-10A cells was most likely due to cell death resulting from telomere damage. Nonetheless, a marked appearance of a Sub-G1 fraction of cells by FACS analysis compatible with apoptosis was not observed after 96 h TRF2^ΔBΔM^ expression (Figure 5A).

What is more, the response of several proteins involved in the DDR was considerably reduced in cells constitutively expressing TRF2^ΔBΔM^ during 96 h when compared to cells treated with the DSB-inducer Bleocin™ (Figure 1C and Supplementary Figure 3A). Monoubiquitination of H2AX (ub-H2AX), which functions as a proximal regulator in the DDR by initiating the DNA damage signalling through efficient γ-H2AX formation [49] was prominent in TO, SH-TO and SV-TO cells exposed to Bleocin™. By contrast, in DOX treated TO and SH-TO cells there was a reduced level of ub-H2AX. Only SV-TO cells showed a marked higher level of ub-H2AX, which could be related to the focal accumulation of γ-H2AX associated with SV40LT antigen infection [50]. Furthermore, expression of p53, p53^S15^ and p21^Waf1/Cip1^ followed the same trend, with a higher induction in Bleocin™ treated cells. In the face of telomere dysfunction induced by TRF2^ΔBΔM^ expression, neither p53 nor p53 phosphorylation and p21^Waf1/Cip1^ upregulation could be detected in TO and SH-TO cells. As a whole, no evidence that 96 h of telomere dysfunction precipitates a DDR in the p16^INK4a^-deficient MCF-10A cells was found. This low level of DNA damage elicited after TRF2 depletion could be the reason for the ambiguous fate of cells carrying telomere damage.

### Even transient cycles of 24 h acute telomere dysfunction do not result in massive CIN

Given that acute telomere dysfunction for 96 h impacted on the development of CIN and the growth rate of cells with telomere damage, we investigated whether shorter periods of telomere deprotection would allow the generation of endless BFB cycles and massive CIN.

**Figure 7:**
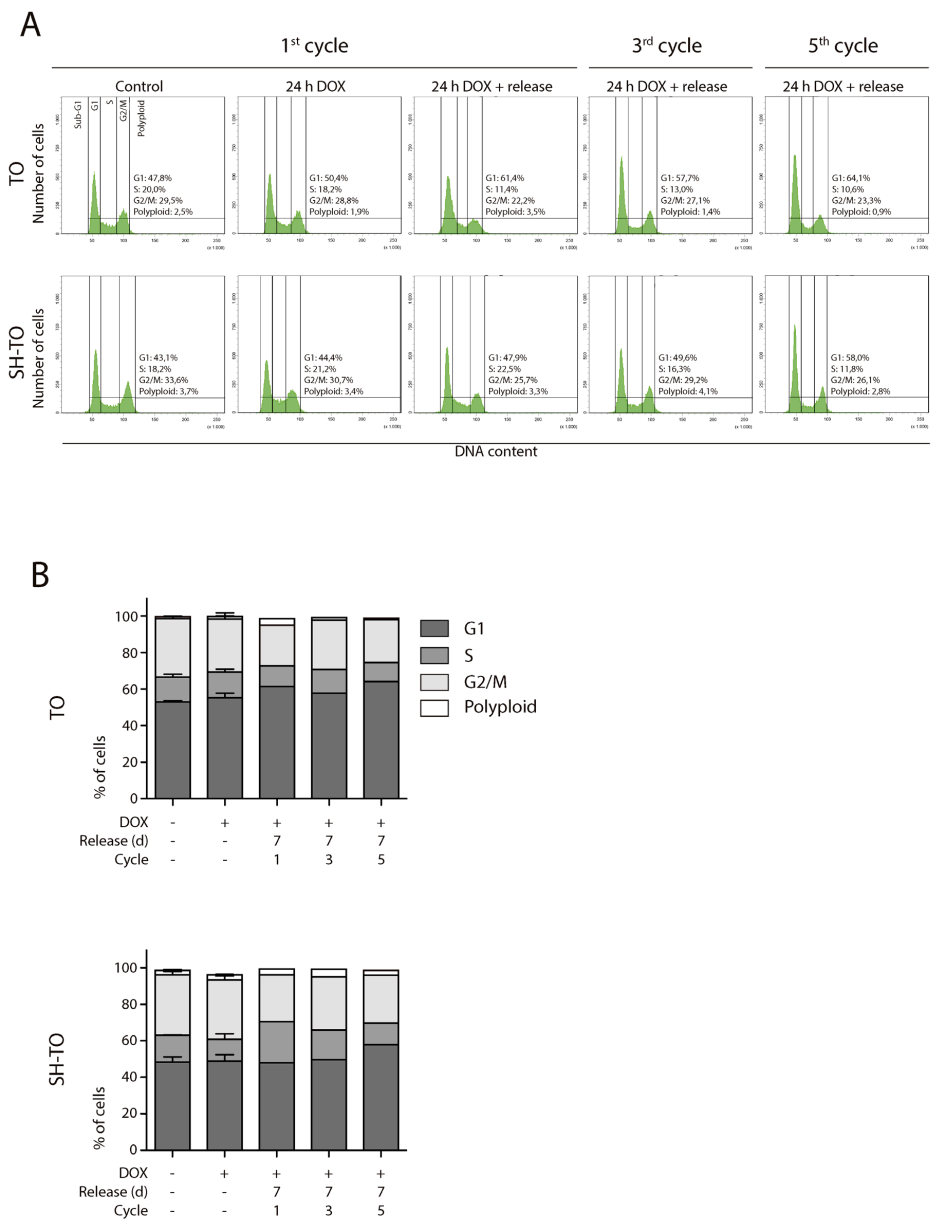
Short TRF2^ΔBΔM^ expression periods did not alter the cell cycle profile **(A)** Representative cell cycle profiles of TO and SH-TO cell lines after periods of short TRF2^ΔBΔM^ expression. The first column shows the control cells. Subsequent columns show 24 h of sustained DOX treatment, 24 h of DOX treatment and 7 days of release, from the first, third and fifth DOX cycle, respectively. Cell cycle profiles remain stable during 24 h of DOX treatment, as well after their release during five cycles of TRF2^ΔBΔM^ expression. Cell cycle phases are marked and values indicated. **(B)** Cell cycle phases distribution among TO and SH-TO cell lines after 24 h DOX exposure experiments. No statistical differences were observed between control and DOX samples treated during 24 h (Mann-Whitney test). Data are presented as mean + SEM from three independent experiments, except for 24 h of DOX treatment and release of the first, the third and the fifth cycles, in which only one experiment is shown. A minimum of 10,000 cells were analysed per experiment.

**Figure 8:**
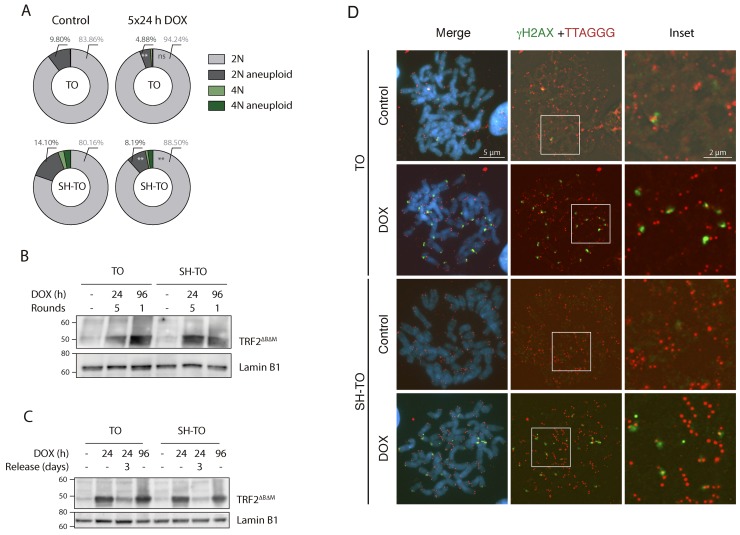
Short cycles of TRF2^ΔBΔM^ expression do not generate unstable cells **(A)** Reduction in 2N-aneuploid cells in TO and SH-TO cell lines when repeated TRF2^ΔBΔM^ expression periods were induced (p= 0.0064 and p= 0.0036, respectively). **(B)** Immunoblot of TRF2^ΔBΔM^ expression in TO and SH-TO cells denotes that the expression of TRF2^ΔBΔM^ was not diluted over time. Protein levels during the fifth 24 h DOX treatment were similar to those observed after 96 h of sustained acute telomere deprotection. Lamin B1 was used as loading control. **(C)** Immunoblot of TRF2^ΔBΔM^ expression after 24 h or 96 h of sustained DOX treatment and after 3 days of recovery from 24 h DOX in TO and SH-TO cell lines. Note that 24 h of DOX treatment was enough to induce the expression of TRF2^ΔBΔM^ and protein levels were similar between 24 h and 96 h of sustained TRF2^ΔBΔM^ expression. Three days after DOX removal, TRF2^ΔBΔM^ was still present but to a lesser extent. Lamin B1 was used as loading control. **(D)** Representative images of γH2AX *foci* (green) and telomere (red) PNA hybridisation in metaphase TO and SH-TO cells. TIFs were observed in both cell lines after 24 h of sustained DOX treatment. Insets indicate colocalisation of γH2AX *foci* and telomere DNA. Scale bar corresponds to 5 μm in the images and to 2 μm in the insets.

Both TO and SH-TO cells were exposed to successive 24 h periods of DOX treatment and washout. Cells retrieved after one or further 24 h DOX periods showed also an unaltered cell cycle profile (Mann-Whitney U-test; p> 0.05) (Figure 7A and 7B). Moreover, cells that received five cycles of 24 h DOX and recovery retained the parental MCF-10A karyotype. Chromosome aberrations in the form of end-to-end fusions were observed, but no signs of ongoing BFB cycles were present in metaphase cells of TO or SH-TO after five 24 h DOX cycles. In addition, oligoFISH analysis revealed a significant reduction in aberrant cells after the fifth 24 h DOX cycle compared to uninduced matched cells (p= 0.0244 and p= 0.0006 for TO and SH-TO, respectively) (Figure 8A). In view of these results, expression of TRF2^ΔBΔM^ was evaluated in those cells subjected to the fifth doxycycline cycle to determine whether the unperturbed cell cycle profile and the decreased frequency of aneuploid configurations, at that time, were due to an increase outgrowth of cells with diminished TRF2^ΔBΔM^ expression. However, this was not the case, as western blot analysis demonstrated that cells retained TRF2^ΔBΔM^ expression during the fifth cycle of 24 h DOX in much the same way that cells express TRF2^ΔBΔM^ after 96 h of doxycycline (Figure 8B and Supplementary Figure 3C). Then, we verified that 24 h of DOX exposure were sufficient to induce TRF2^ΔBΔM^ expression and that, after DOX washout, TRF2^ΔBΔM^ levels were reduced (Figure 8C and Supplementary Figure 3C). Finally, the efficacy of 24 h of DOX exposure in promoting uncapped telomeres was revealed by the presence of primary end-to-end fusions and telomere dysfunction induced *foci* (TIFs) in mitotic cells (Figure 8D).

Together, these results somehow support the notion that p16^INK4a^-deficient MCF-10A cells, even in an impaired p53 background, cannot withstand short periods of acute telomere damage and are designated to stop proliferation and die.

## DISCUSSION

In human primary cells replication-dependent telomere attrition leads to the accumulation of dysfunctional telomeres, which have been recognised to play a relevant role in controlling the proliferative boundaries and fate of human cells. It is known that cells containing fewer than five dysfunctional telomeres can proliferate without halting the cell cycle [51], but above this threshold, cells enter senescence. Hence, the natural ends of chromosomes function as tumour suppressors by limiting the outgrowth of incipient tumour cells. At this point, full deprotection of telomeres is not occurring, as end-to-end chromosome fusions are not observed [51]. Circumvention of this growth arrest, in most human cells, needs combined inactivation of p53 and pRb pathways. Strikingly, primary HMECs cultured *in vitro* can avoid the M1 growth arrest by spontaneous silencing of the *CDKN2A* gene through promoter hypermethylation [52, 53]. In the absence of p16^INK4a^ expression, HMECs acquire an extended lifespan, where further telomere shortening results in the progressive transit of telomeres from a closed state to an uncapped state. As human telomeres are heterogeneously sized, physiological telomere erosion in vHMECs leads to the gradual appearance of unprotected telomeres that are continuously repaired by fusing with each other [5]. This reduces the initial damage and allows massive remodelling and scrambling of the genome through endless BFB cycles on proliferating cells (reviewed in [43]). Nevertheless, these proliferating unstable cells finally succumb to p53-dependent growth arrest, called agonescence, or crisis if p53 function is abrogated [54]. It is thought that stabilisation of telomere length in these genome unstable cells would alleviate DNA damage and rescue cellular fitness at a cost of driving to malignancy.

With the aim of generating immortal mammary cells that have passed through a period of telomere instability, we have set up a reversible system of acute telomere deprotection by controlling the expression of TRF2^ΔBΔM^ in the p16^INK4a^-deficient MCF-10A breast epithelial cell line. We hypothesised that transient periods of telomere dysfunction through shelterin modification in telomerase proficient cells would drive BFB cycles and produce some degree of ongoing instability at a level compatible with cell viability. Our study demonstrates that controlled TRF2 depletion efficiently produced a telomere dysfunction phenotype in the modified MCF-10A cell lines. Nevertheless, we did not find clear evidence of ongoing BFB cycles, such as secondary chromosome aberrations with interstitial telomeric DNA sequences, increased aneuploid configurations in interphase nuclei or the accumulation of cells with 8C DNA content compatible with cycling polyploids. Despite the fact that the increased fraction of anaphase cells with chromatin bridges could reflect cell cycle progression, the lack of telomere-dependent CIN somehow denotes the absence of long term proliferation of cells with telomere damage. Indeed, reduced cell growth was discernible in the modified cell lines exposed to TRF2^ΔBΔM^. But the proliferation defects were not accompanied by cell cycle disturbances or visible markers of senescence or apoptosis. What is more, 96 h of telomere dysfunction did not precipitate a DDR comparable to the one elicited by the DSBs-inducer Bleocin™. The low level of DNA damage inflicted after TRF2 depletion could be the reason for the ambiguous disappearance of cells carrying telomere damage. Thus, it seems likely that TRF2^ΔBΔM^ expression induces proliferation defects in p16^INK4a^-deficient MCF-10A breast epithelial cells that conclude in their clearance from the cell culture, presumably because of a telomere capping defect rather than cell death associated with telomere-dependent extensive genome instability.

In contrast to progressive telomere shortening, the expression of TRF2^ΔBΔM^ suddenly evokes the direct transition of several telomeres from a closed to a deprotected state [27, 55]. In primary fibroblasts, the cellular response to TRF2^ΔBΔM^ expression somehow recapitulates telomere-dependent replicative senescence, but cells stop division, displaying telomere-to-telomere fusions, chromatin bridges at anaphase, and nearly tetraploid karyotypes [20, 22, 23]. The growth arrest imposed by TRF2 deletion in primary fibroblasts is overridden by combined inactivation of p53 and pRb pathways through SV40LT [20] or HPV16 E6E7 [56] infection. Also, when p16^INK4a^ inhibition is combined with p53 inactivation, a nearly complete bypass of telomere-induced senescence is achieved [48]. Even though, the telomere damage inflicted results, in all instances, in lethal genome instability [20, 48, 56]. Unlike fibroblasts, the proliferation restraint elicited by sustained TRF2^ΔBΔM^ expression in p16^INK4a^-deficient MCF-10A cells was not circumvented by short hairpin p53 inactivation, thus revealing a distinct cellular response of the mammary epithelial lineage to TRF2 depleted telomeres. Such cell lineage discrepancy was probably not related to the intensity of the telomere damage inflicted, as the rate of cells containing end-to-end fusions and the frequency of end fusions per cell were similar between the Jacobs and de Lange study and the results presented herein [48]. The most likely interpretation is that epithelial cells showing p16^INK4a^ deficiencies are highly sensitive to acute DNA damage derived from sustained TRF2^ΔBΔM^ expression and are committed to stop proliferating and probably die. This assumption is also reinforced by the presence of meta-TIFs after 24 h TRF2^ΔBΔM^ expression and by the karyotype analysis of cells retrieved after transient periods of short TRF2^ΔBΔM^ expression. The reduction in the telomere insult to shorter periods again demonstrated a lack of hallmarks compatible with ongoing BFB cycles. Altogether, these data suggest that even brief TRF2 depletion periods in p16^INK4a^-deficient MCF-10A cells leads to a level of telomere damage that is incompatible with cell proliferation, and supports the view that the telomere insult itself, and not the genomic instability associated with BFB cycles, is responsible for the deleterious effects on cell proliferation [57].

In summary, the severity of the cellular responses to progressive or acute telomere dysfunction are not analogous among mammary epithelial cells and seems to be dependent on the severity of the telomere damage impinged. p16^INK4a^-deficient breast epithelial cells react to the minor damage of progressive telomere uncapping by stimulating repair and cell survival at a cost of unleashing genome instability through the onset of BFB cycles [43]. Conversely, TRF2-depleted telomeres in p16^INK4a^-deficient mammary MCF-10A cells results in a proliferative block that prevents the generation of genome unstable cells. This halt in cell cycle progression is not due to the sustained telomere damage, as even transient cycles of short TRF2 deprotection were unable to drive chromosome instability. We propose that the deprotection of many telomeres simultaneously, above a certain DNA damage threshold, probably results in a local activation of the cellular stress response that consequently triggers cell withdrawal from cell cycle to maintain genomic integrity.

## MATERIALS AND METHODS

### Cell lines

Non-transformed human mammary epithelial MCF-10A cells, provided by Dr Carme Nogués, and derived cell lines were cultured in DMEM:F12 (GIBCO, ThermoFisher Scientific) supplemented with 2% tetracycline-free horse serum (GIBCO), cholera toxin (100 ng/ml) (Sigma-Aldrich), hEGF (20 ng/ml) (Sigma-Aldrich), hydrocortisone (0.5 μg/ml) (Sigma-Aldrich), insulin (10 μg/ml) (Sigma-Aldrich) and 1% penicillin/streptomycin (GIBCO). Post-stasis variant human mammary epithelial cells (vHMECs) were obtained from Cell Applications Inc. (San Diego, CA, USA). Pre-stasis HMECs were established from reduction mammoplasty tissue in accordance with previously reported methods [58]. The patient signed a written consent form allowing their tissue to be used for biological research; this consent was obtained by the medical staff at the hospital prior to surgery. All work with human derived material was reviewed and approved by the Human Subjects Protection Committee of the Universitat Autònoma de Barcelona. vHMECs and HMECs were cultured with serum-free MEpiCM medium supplemented with MEpiCGS and penicillin/streptomycin (all from ScienCell Research Laboratories, Carlsbad, CA, USA), or with M87AX [59]. HEK 293T cells were cultured in DMEM (GIBCO) supplemented with 10% of foetal bovine serum (GIBCO) and 1% of penicillin/streptomycin.

Growth conditions were 5% CO_2_ and 37°C. Culture population doublings (PDs) were calculated using the formula: PD = PD_initial_ + log_2_ (N_final_/N_initial_), where N_initial_ is the number of viable cells plated, and N_final_ is the number of viable cells harvested.

### Lentiviral vectors

A lentiviral tetracycline-inducible TRF2^ΔBΔM^ construct was generated by cloning the inducible TRF2^ΔBΔM^ cassette from a pBluescript.KS vector (courtesy of Dr Lenhard Rudolph) into a neomycin resistant promoter-less lentivector (Amsbio) using X-baI restriction sites. The Tet-regulated transcriptional transactivator protein rtTA3 containing hygromycin resistance was a kind gift from Dr Iain Fraser. The lentiviral construct for p53 short hairpin RNA (shp53 pLKO.1 puro) was from Dr Bob Weinberg (Addgene plasmid #19119), and the lentiviral plasmid for SV40LT (pRRLsin-SV40 T antigen-IRES-mCherry) was from Dr Snorri Thorgeirsson (Addgene plasmid #58993). The hTERT lentivirus was supplied by Viral Vector Facility, CNIC, Spain.

### Lentivirus production and transduction

To generate lentiviral particles, the psPAX2 and pMD2.G plasmids together with the plasmid containing the gene of interest were introduced in HEK 293T packaging cells using Calphos Mammalian Transfection kit (Clontech). Supernatants were collected at 48 and 72 h post-transfection and concentrated using Amicon 100,000 centrifugal filter units (Merck-Millipore).

The MCF-10A T/O TRF2^ΔBΔM^ (TO) cell line was generated by lentiviral transduction of MCF-10A cells with the TRF2^ΔBΔM^ inducible cassette and the rtTA3 transactivator, with polybrene (4 μg/ml) (Sigma-Aldrich). Transduced cells were selected first with G418 (300 μg/ml) (Sigma-Aldrich) and afterwards with hygromycin (300 μg/ml) (Sigma-Aldrich). The MCF-10A T/O TRF2^ΔBΔM^-SHP53 (SH-TO) was generated by lentiviral transduction of shp53 and selection with puromycin (0.75 μg/ml). The MCF-10A T/O TRF2^ΔBΔM^-SV40LT (SV-TO) cell line was generated by transduction of MCF-10A T/O TRF2^ΔBΔM^ with SV40LT-mCherry lentiviral particles. Selection of the different transduced cells was performed through one week culture with the appropriate antibiotic or, in the case of SV-TO cells, through FACS sorting of mCherry positive cells.

vHMECs expressing hTERT (vHMEC-hTERT) were generated by lentiviral transduction of vHMECs with the hTERT lentivirus at PD 21. HMECs-hTERT expressing SV40LT were generated by lentiviral transduction of SV40LT-mCherry lentiviral particles at passage 2 (PD 6.92).

### Expression of TRF2^ΔBΔM^

Induction of telomere dysfunction in the different conditional cell lines was performed by culturing the cells with regular MCF-10A medium containing 1 μg/ml doxycycline (DOX) (Sigma-Aldrich). Long-term telomere dysfunction experiments consisted of the persistent exposure of cells to DOX for 96 h. In all experiments, fresh DOX was added to the cell culture each 48 h. Moreover, conditional studies were conducted where telomere dysfunction was switched on and switched off by the addition/removal of DOX from the culture medium. Long-term exposure cycles consisted of 96 h of sustained TRF2^ΔBΔM^ expression and 3 days without DOX, whereas in short-term cycles, cells where treated with DOX for 24 h and then were exposed to DOX-free medium for 7 days.

### Western blotting

Proteins were extracted with 2% SDS, 67 mM Tris HCl (pH 6.8) or RIPA lysis buffer, containing protease and phosphatase inhibitors. Protein extracts were sonicated twice at 25% amplitude for 15 s, boiled at 95°C for 15 min and centrifuged at 20,000 g for 10 min. Proteins were quantified using the BCA method and absorbance was read at 540 nm with a Victor3 spectrophotometer (PerkinElmer). The proteins (30 μg) were separated using 3-8% Tris-acetate or 10% Bis-Tris gels (Life Technologies, ThermoFisher Scientific) at 35 mAmp and transferred onto nitrocellulose or PVDF membranes at 30 V. Membranes were blocked with 5% non-fat milk or BSA. Primary antibodies used were: mouse anti-SV40 st+LT Ag (Santa Cruz; sc-148), rabbit anti-pRb^S807/811^ (Cell Signaling; D20B12), mouse anti-pRB (Cell Signaling; 4H1), rabbit anti-p53^S15^ (ThermoFisher Scientific, 14H61L24), mouse anti-p53 (ThermoFisher Scientific, DO-1), mouse anti-γH2AX (Upstate; 07-164), rabbit anti-p21^Waf1/Cip1^ (Cell Signaling; 12D1) and mouse anti-TRF2 (Novus Biologicals; 4A794.15). Furthermore, mouse anti-α-Tubulin (Sigma, B-5-1-2) and rabbit anti-lamin B1 (Abcam; ab16048) were used as loading controls. Primary antibodies were incubated overnight at 4°C. Secondary anti-mouse or anti-rabbit horseradish peroxidase (HRP) conjugated antibodies were used and incubated for 1 h at room temperature. Chemiluminescent detection was performed using HRP solution and luminol (Life Technologies), and images were acquired using Chemidoc, processed with Quantity One software and analysed with ImageLab™ 6.0.0 (BioRad).

### Drug treatments

DSBs were generated in MCF-10A and derivatives through exposure to the radiomimetic drug Bleocin™ (Calbiochem, Merck-Chemicals; Germany), a bleomycin compound, at a final concentration of 2.5 μg/ml. The drug was washed out after 1 h exposure and the cells were left to recover for 60 min before protein extraction.

Colcemid (GIBCO) at a final concentration of 50 ng/ml was added to asynchronously proliferating TO, SH-TO and SV-TO cells. After 24h of colcemid exposure, the cells were collected and fixed in 70% ethanol and kept frozen until FACS processing. Additional experiments consisted of 24 h colcemid treatment, washout and 24 h or 48 h release before fixation.

### Obtaining metaphase cells and end-to-end fusion scoring criteria

Exponentially growing MCF-10A and, untreated and doxycycline-treated MCF-10A modified cell lines were exposed to colcemid (0.5 μg/ml) for 2 h. Cells were trypsinised, swollen in 0.075 M KCl and fixed in methanol:acetic acid (3:1). Cell suspensions were dropped onto clean slides and stored at -20°C until use. For end-fusion scoring purposes, slides were first stained with DAPI. Then, the metaphase plates were captured and the karyotype was performed by reverse DAPI staining, which results in a reproducible G band-like pattern that allows for accurate individual chromosome identification before the chromosomes became swollen by the denaturation step. Afterwards, the slides were hybridised with the PNA probes and the metaphases were relocated to analyse the telomere and centromere status of each chromosome. A fusion event was considered when the connection between chromatids (1 or 2) was verified on the initial DAPI stained image. This procedure reduces the possibility of end-fusions events being confused with mere alignment of chromosomes.

### In situ fluorescence hybridisation

#### Telomere and centromere PNA-FISH

Metaphase spreads were hybridised with pantelomeric (Rho-(CCCTAA)_3_, PE Biosystems) and pancentromeric (FITC-AAACACTCTTTTTGTAGA, Panagene) PNA probes. Denaturation took place at 80°C for 3 min and hybridisation was performed at 37°C for 2 h in a humid chamber. Afterwards, slides were washed twice with 70% formamide for 15 min, followed by three TNT (Trizma Base 50 mM, NaCl 150 mM and Tween20 0.25%) washes for 5 min. Dehydrated slides were counterstained with DAPI.

#### OligoFISH

Interphase nuclei spreads were treated with pepsin-HCl at 37°C for 10 min, post-fixed with formaldehyde-MgCl_2_ and denatured with 70% formamide at 74°C. Specific centromeric probes for chromosomes 6 (Gold DY539), 12 (Red DY590) and 17 (Green DY490) (Cellay, Inc.) were hybridised for 2 h in a humid chamber followed by one 5 min wash with 0.2xSSC-0.1%SDS at 50°C and a 2xSSC wash. Cells were dehydrated and counterstained with DAPI.

### DAPI staining, immunofluorescence and TIFs (telomere dysfunction induced *foci*)

For anaphase bridge scoring, cells cultured on coverslips were fixed with 4% paraformaldehyde (PFA) at 37°C, rinsed twice in PBS, allowed to dry and counterstained with DAPI.

For immunofluorescence, cells were fixed in 4% PFA for 10 min at 37°C, permeabilised with 1% Triton X-100 at room temperature and blocked with 5% FBS-0.1% Triton X-100- KCM buffer. Then SV40 st+LT Ag (1:200) primary mouse antibody was incubated overnight at 4°C. Conjugated chicken Alexa Fluor 488-antimouse antibody (1:500, ThermoFisher Scientific) was incubated for 1 h. Cells were counterstained with DAPI.

For the TIF assay, cells were treated with DOX for 24 h. The following day, chromosome spreads were obtained as described by [60]. Briefly, after 25 min of colcemid (20 ng/ml), the cells were trypsinised and resuspended to a final concentration of 5x10^4^ cells/ml in hypotonic solution (0.2% Trisodium Citrate-0.2% KCl). Thereafter, the cells were cytocentrifuged 10 min at 1,000 rpm and the slides were air dried. Afterwards, the slides were treated with pre-extraction buffer for 5 min, 1% Triton X-100- KCM buffer for 10 min and fixed with 4% PFA for 10 min. Permeabilisation was also performed with chilled absolute methanol during 20 min. Cells were blocked with 2% FBS-0.1% Triton X-100- 100 μg/ml RNAse- TrisNaCl for 30 min at 37°C, and mouse anti-γH2AX (1:500, clone JBW301 Millipore) antibody was incubated at 4°C. Conjugated chicken Alexa Fluor 488-antimouse was incubated for 1 h. Slides were fixed again in 2% PFA for 10 min and dehydrated through an ethanol series. Samples and pantelomeric probe (Rho-(CCCTAA)_3_, PE Biosystems) were co-denatured at 80°C for 4 min, and hybridised overnight at 37°C in a humid chamber. Slides were washed once with 70% formamide-Tris and twice with PBST. To ensure γH2AX staining, conjugated Alexa Fluor 488-antimouse was re-incubated for 30 min. Cells were dehydrated and counterstained with DAPI.

### Fluorescent microscopy and fluorescent images

Most fluorescent staining was visualised under an Olympus BX60 microscope equipped with epifluorescent optics and a camera (Applied Imaging, Inc.). In the case of TIFs analysis, an Olympus BX61 epifluorescence microscope with motorized x-y stage (BX-UCB, Olympus) was used to acquire images as a Z-stack (total of 7 planes of 2.11 μm each). The fluorochromes were visualised through simple filters and images were captured and analysed using Cytovision software (Applied Imaging, Inc.).

### Flow cytometry and live cell sorting

For tracking S phase cells, a pulse of 10 μM BrdU was carried out for 30 minutes. Afterwards, the cells were rinsed, trypsinised, centrifuged and fixed with 70% ethanol. For cell cycle analysis or SV40 st+LT detection, the cells were harvested and fixed in 70% ethanol and kept at -20°C until processing.

For SV40 st+LT and BrdU analysis, the fixed cells were permeabilised with 1xPBS-1%Triton X-100 solution. In the case of BrdU detection, DNA was denatured with HCl 2N-0.1% Triton X-100. After 30 min, denaturation was blocked by adding tetraborate solution (0.1 M). Before antibody detection, every sample was divided into two tubes, one for mouse anti-SV40 st+LT Ag (Santa Cruz; sc-148) or mouse anti-BrdU (Santa Cruz, sc32323) and subsequent Alexa Fluor 488-antimouse secondary antibody. The second tube was used as a negative control of the secondary antibody.

All cells were stained with propidium iodide solution (PBS-1% Triton X-100, propidium iodide 45 μg/ml, and RNase 0.2 mg/ml) before cytometric processing. Analysis was performed under a FACSCalibur (Beckton Dickinson). Sample excitation was done with a 488 nm laser and a minimum of 10,000 events were collected per sample. Single cells were gated first by forward scatter (FSC) and side scatter (SSC), and DNA content of single cells was measured on FL3 (670 nm long pass filter) and plotted vs. number of cells. Alternatively, A488 fluorescence was detected on FL1 (530/30 nm band pass filter) and was plotted vs. propidium iodide. The data were analysed with BD FACSDiva software v7.0.

For live cell experiments, cells were trypsinised and resuspended in PBS. Sorting of mCherry positive cells was carried out using a FACSAria I SORP sorter (Beckton Dickinson). Excitation of the sample was done using a 488 nm laser for FSC and green fluorescence parameters, and a 561 nm laser was used for the excitation of mCherry and SSC signals. Cells were gated according to their FSC vs. SSC parameters. Red emission from mCherry (610/20 nm) excited with the yellow laser (561 nm) was plotted vs. green emission (530/30 nm) from the 488 nm laser on a dot plot in order to discriminate mCherry positive cells.

### Senescence associated beta-galactosidase staining

TO and SH-TO cells treated with DOX (1 μg/ml) for 24 h and 96 h and left to recover until the seventh day were seeded at a concentration of 7,500 cells/well. A positive control consisted of cells treated with Bleocin™ (2.5 μg/ml) for 24 h and left to recover until the seventh day. In Bleocin™ experiments 50,000 cells/well were seeded. The Senescence β-Galactosidase Cell Staining Kit (Cell Signaling) was used according to the manufacturer’s instructions. The cells were examined under a light microscope (Olympus IX71) and those cells with blue staining were considered positive for β-galactosidase activity. We estimated the percentage of β-galactosidase staining by analysing 5 individual fields per well from three replicates.

### Statistical analysis

Data analysis was carried out with GraphPad Prism version 5 software (GraphPad Software Inc.). Normality distribution was tested by Saphiro-Wilk normality test. Data sets were compared using Fisher’s exact test, Mann-Whitney test, Kruskal-Wallis test and Dunn’s multiple comparison post-test. P-values less than 0.05 were considered significant. When multiple comparisons were done, the Bonferroni p-value correction was applied and indicated.

## SUPPLEMENTARY MATERIALS FIGURES AND TABLES



## Abbreviations

ALT-pathway: Alternative Lengthening of Telomeres pathway
BFB: Breakage Fusion Bridge
BrdU: 5-bromo-2’deoxyUridine
CDKN2A: Cycling Dependent Kinase Inhibitor 2A
CIN: Chromosome INstability
DAPI: 4′,6-diamidino-2-phenylindole
DCIS: Ductal Carcinoma *In Situ*
DDR: DNA Damage Response
DOX: Doxycycline
DSB: Double Strand Break
hEGF: human Epidermal Growth Factor
HMEC: Human Mammary Epithelial Cell
NRT: Non Reciprocal Translocation
PD: Population Doubling
Rho: Rhodamine
TIF: Telomere-dysfunction Induced *Foci*
TRF2: Telomere Repeat binding Factor 2
vHMEC: variant Human Mammary Epithelial Cell
