# Supplementary material for: Acute telomere deprotection prevents ongoing BFB cycles and rampant instability in p16INK4a-deficient epithelial cells
Source: Oncotarget. 2018 Jun 5;9(43):27151–70. doi: 10.18632/oncotarget.25502 (PMC6007466; doi:10.18632/oncotarget.25502)
Supplement: Supplementary file 1 [file oncotarget-09-27151-s001.pdf]

Acute telomere deprotection prevents ongoing BFB cycles and rampant instability in p16^{INK4a}-deficient epithelial cells

SUPPLEMENTARY MATERIALS

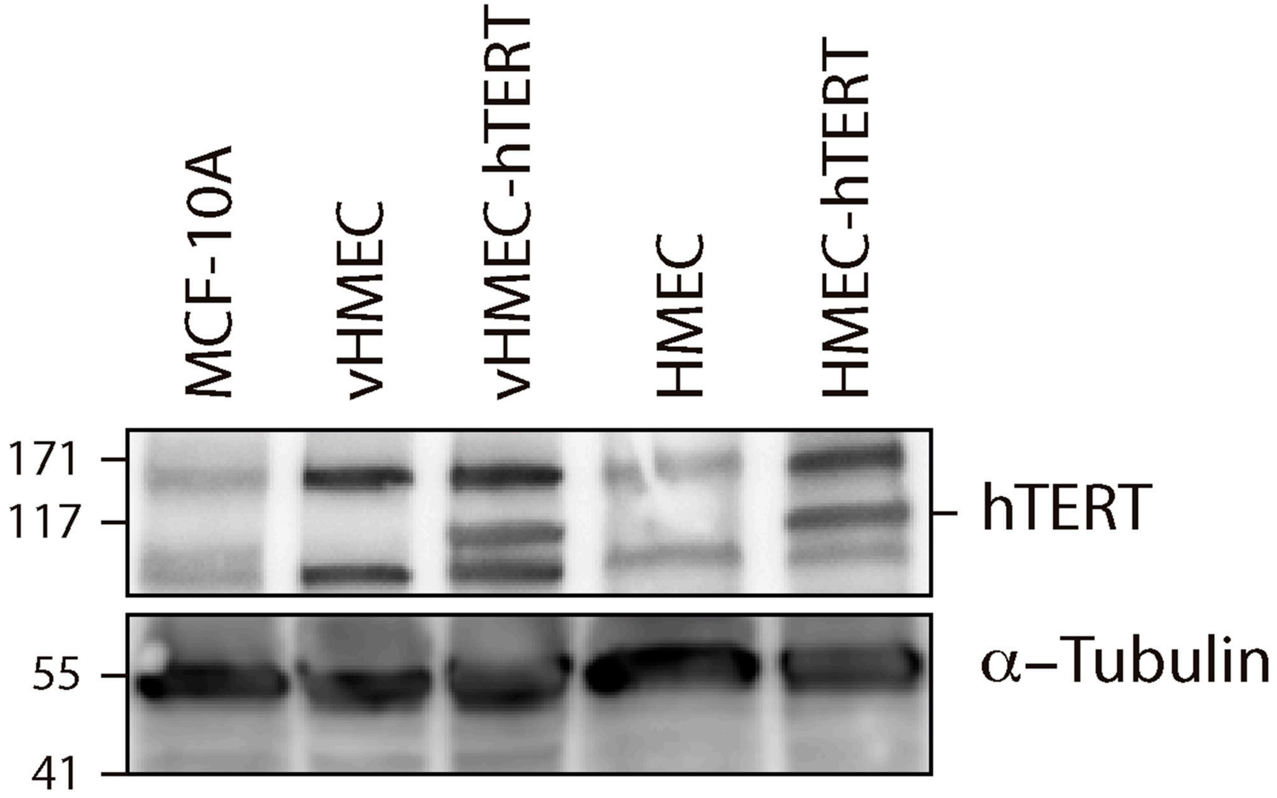

Supplementary Figure 1: hTERT protein levels in MCF-10A cells, as well as vHMECs and HMECs transduced/not transduced with hTERT. Telomerase activity was assessed through expression of the hTERT catalytic subunit. As positive controls vHMECs and HMECs non-transduced and transduced with hTERT lentiviral particles were used. A clear band at approximately 120 kDa was detected in cells transduced with the hTERT lentivirus. MCF-10A protein extracts did not demonstrate a discernible hTERT band.

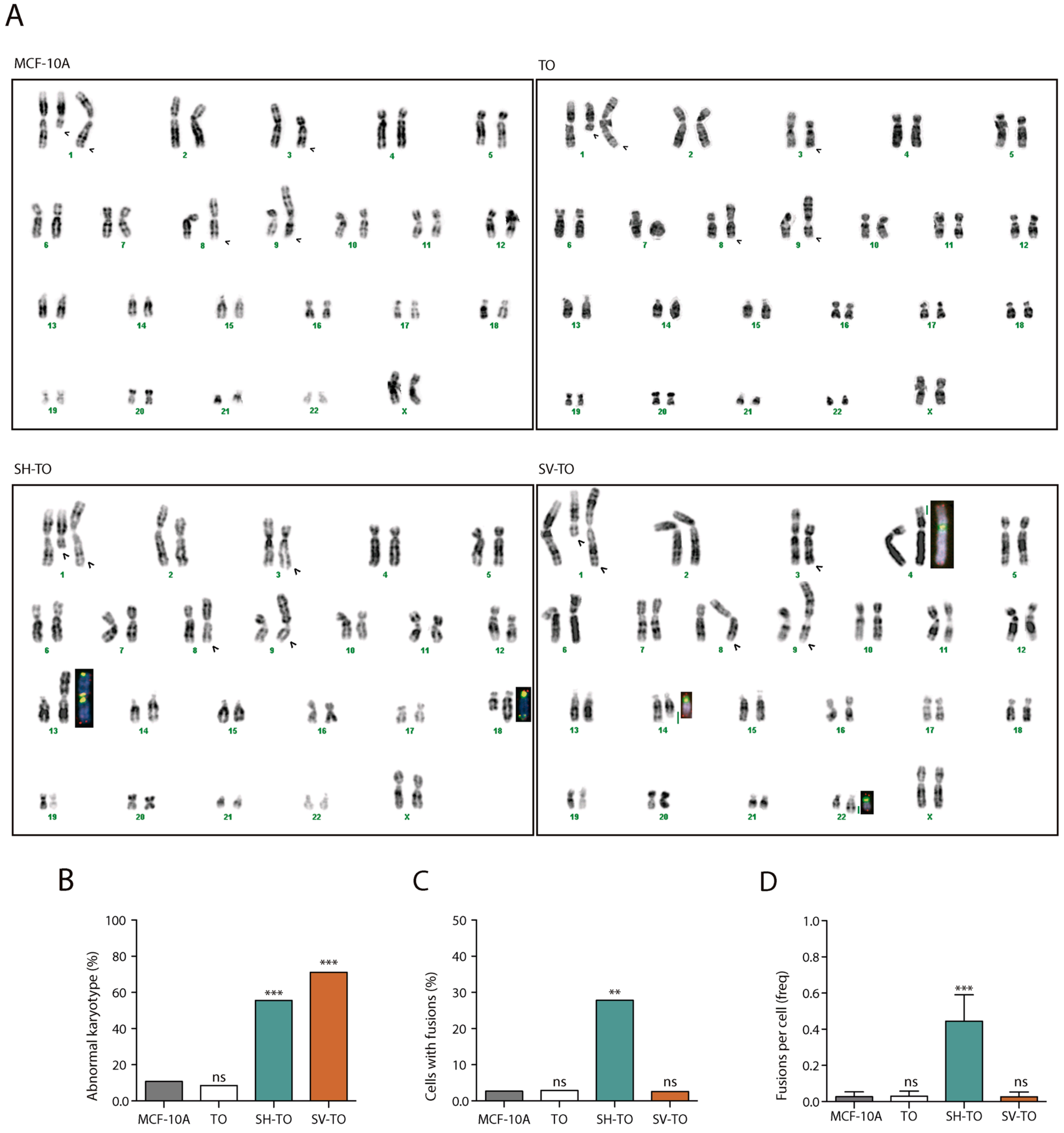

Supplementary Figure 2: Cytogenetic characterisation of MCF-10A and uninduced derivatives. **(A)** MCF-10A; TO; SH-TO and SV-TO representative karyotypes. Open arrows indicate clonal aberrations in the parental MCF-10A cell line. Uninduced TO cells presented the same karyotype as the parental cell line. However, SH-TO and SV-TO cell lines showed additional aberrations. In SH-TO, these consisted mainly of dicentric chromosomes, while non-reciprocal translocations were observed in the SV-TO cell line. Insets in the karyotype show rearranged chromosomes stained with centromeric (green) and telomeric (red) PNA probes. Green lines denote gains or losses. **(B)** Proportion of abnormal karyotypes in each cell line relative to the basal MCF-10A karyotype. No karyotype differences were observed when comparisons were made with uninduced TO cells. In contrast, abrogation of p53 (SH-TO) and p53/Rb pathways (SV-TO) significantly increased the incidence of abnormal karyotypes. No significant differences were observed between SH-TO and SV-TO cells. **(C)** In uninduced cells, the percentage of metaphases containing chromosome fusions was low except in the SH-TO cell line. This reflects that the rearranged karyotype observed in SV-TO cells results from chromosome aberrations other than dicentrics. **(D)** Accordingly, only uninduced SH-TO cells showed a significant increase in the frequency of fusion events per cell. Importantly, most of these fusion events (78.57%, Supplementary Table 2) did not show telomeric FISH signals at the fusion point, thus supporting rejoining of broken DNA ends as origin. Data are presented as mean + SEM.

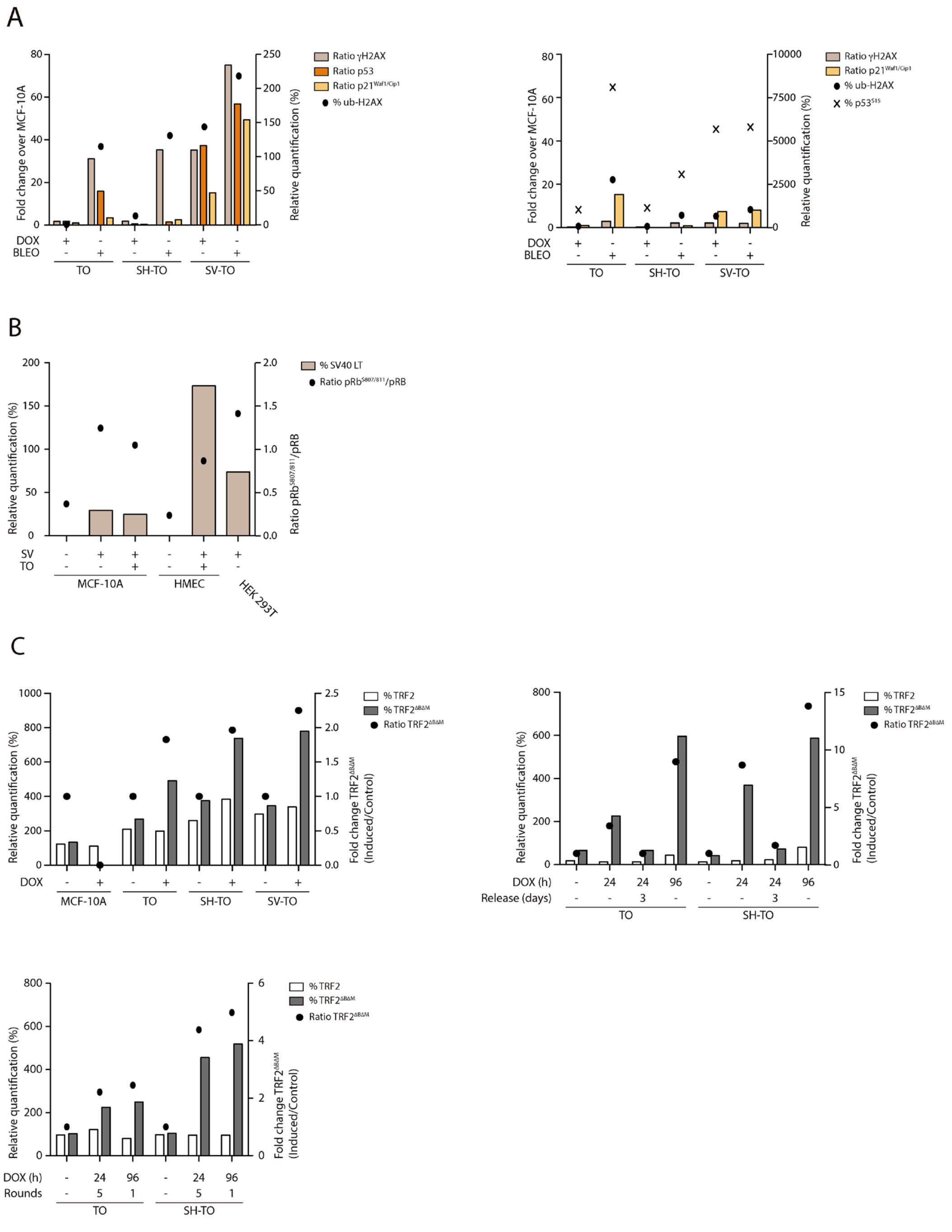

Supplementary Figure 3: Western blot protein quantification. Optical density units of each protein were obtained by ImageLab software. These data were normalised for the loading control Lamin B1 or α-Tubulin. Moreover, additional normalisation was performed by considering protein loading efficiencies. **(A)** The quantification values of ub-H2AX and p53^{S15} were plotted as dots and crosses at the right axis, respectively. The relative abundance of p53, γH2AX and p21$^{Waf1/Cip1}$ in each cell line was compared to the protein levels of untreated MCF-10A cells (not shown), and were plotted as solid bars at the left axis. **(B)** The ratio of pRb$^{S807/811}$ quantification values in relation to the pRb ones were plotted as dots at the right axis. The quantification values of SV40LT was plotted as solid bars at the left axis. **(C)** The quantification values of TRF2 and TRF2$^{\Delta B\Delta M}$ in each cell line were plotted as solid bars at the left axis. The ratio of TRF2$^{\Delta B\Delta M}$ quantification values in the DOX-treated cell lines in relation to the TRF2$^{\Delta B\Delta M}$ of the respective untreated control cells, were plotted as dots at the right axis.

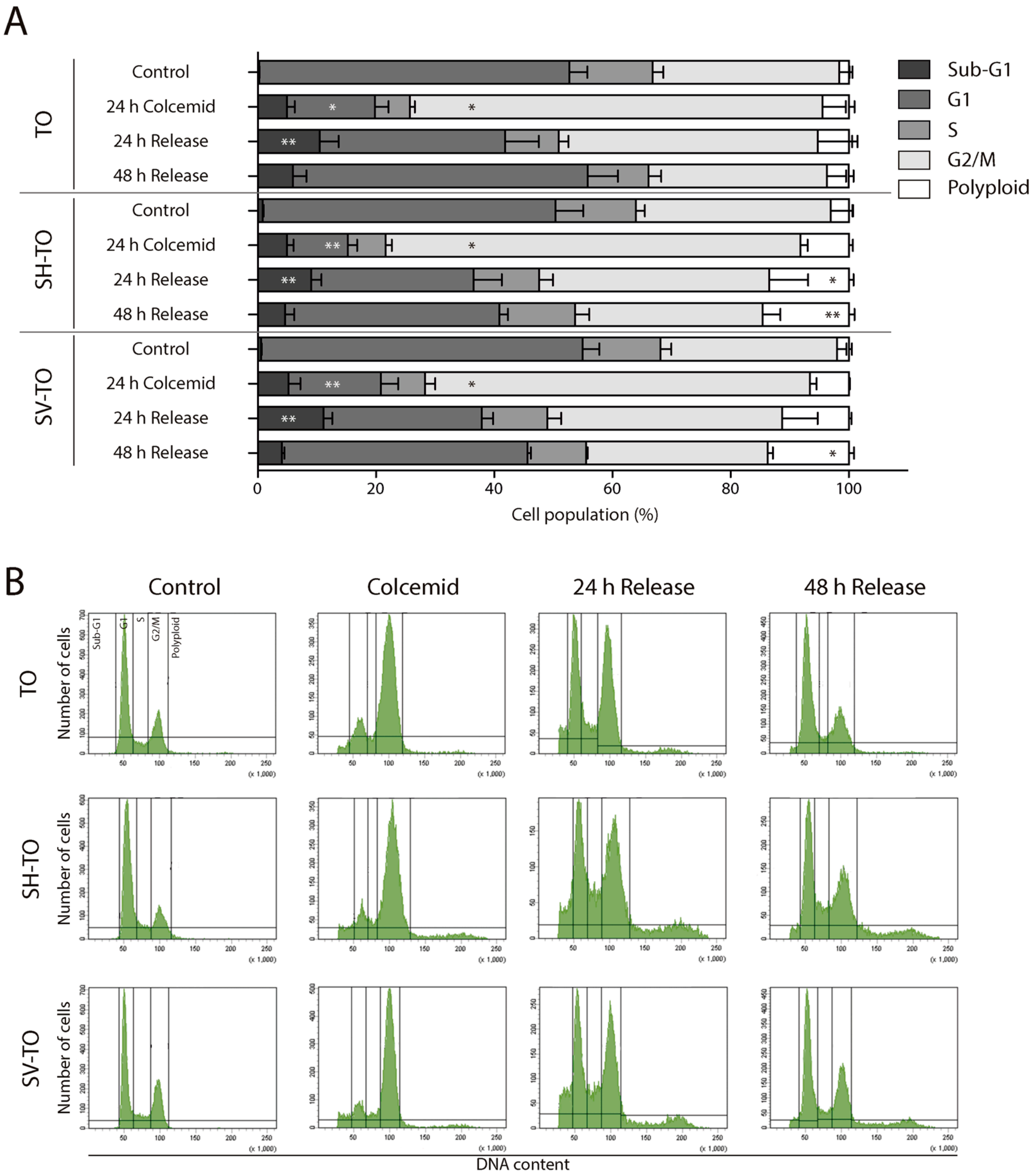

Supplementary Figure 4: Tetraploid checkpoint functional analysis. **(A)** Distribution of cell cycle phases among TO, SH-TO and SV-TO cell lines, in control conditions, treated with colcemid (50 ng/ml) for 24 h, and after a release of 24 h and 48 h. Acute colcemid treatment produced a significant accumulation of cells in G2/M phase that was accompanied by a reduced G1 fraction of cells (Kruskal Wallis and Dunn's comparison test; $p < 0.05$). Cell death was significantly denoted in the following 24 h after colcemid treatment and washout (Kruskal Wallis and Dunn's comparison test; $p < 0.05$). Only in SH-TO and SV-TO cell lines, there was an increased tetraploid population 48 h after colcemid treatment. These data indicate that only SH-TO and SV-TO cell lines allowed the proliferation of tetraploid cells. Data are presented as mean + SEM from three independent experiments. **(B)** Representative plots of cell cycle profiles from each cell line and condition. Cell cycle phases are marked.

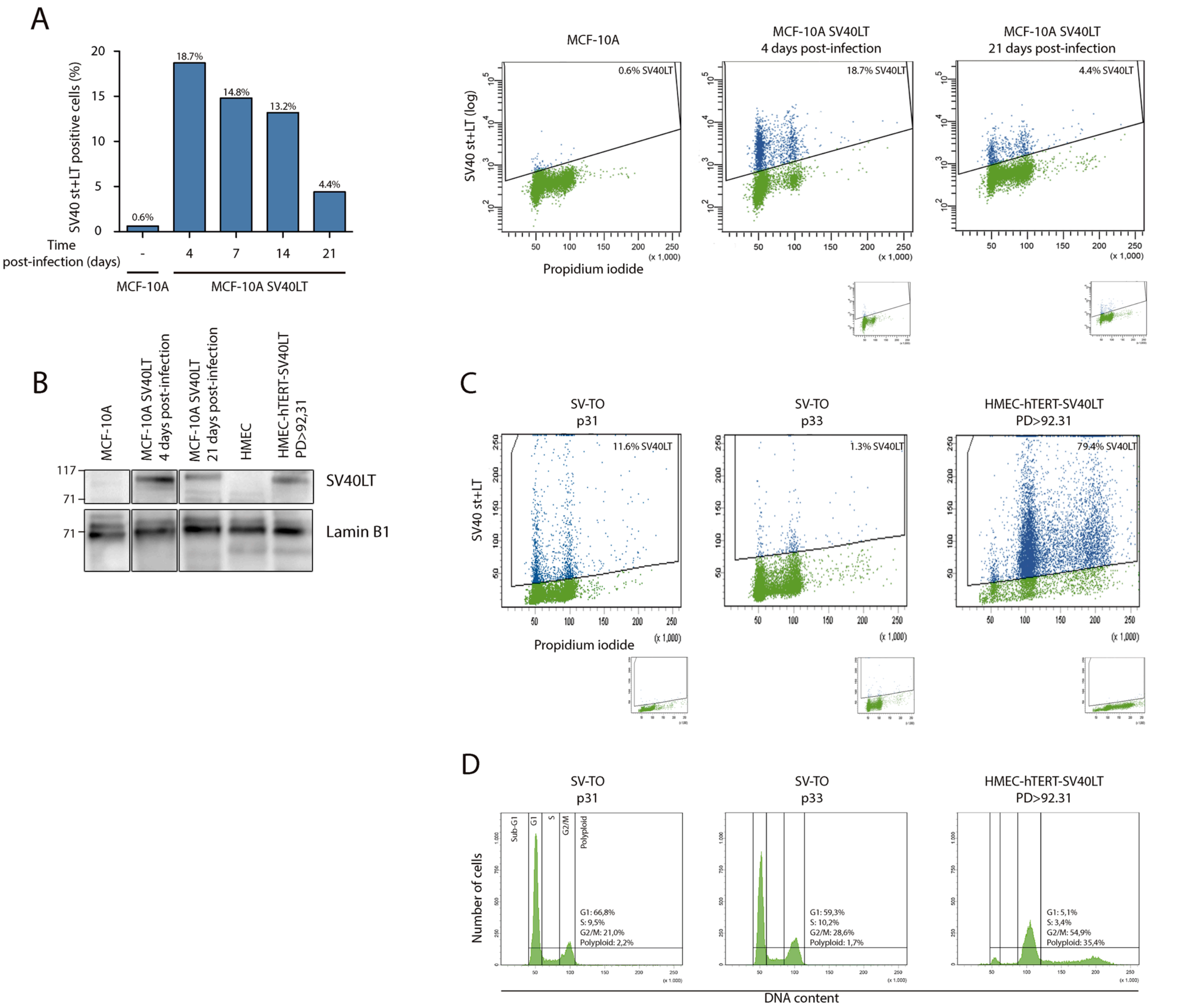

Supplementary Figure 5: Leaking of SV40LT antigens with PDs in MCF-10A. **(A)** MCF-10A cells were infected with SV40LT-mCherry vector and the percentage of SV40LT positive cells was evaluated by flow cytometry using antibodies against SV40 st+LT during three weeks. Four days after infection, 18.7% of cells were positive for SV40 st+LT. This percentage steadily decreased with days in culture. Three weeks after infection, only 4.4% of cells expressed SV40 st+LT antigens. Representative cytometric plots of MCF-10A and SV40LT infected MCF-10A are shown. The absolute percentage of SV40LT cells, where positive cells of the A488 control have been subtracted, is shown. The respective control A488 plots are the smaller ones depicted. **(B)** Immunoblot of control MCF-10A, MCF-10A infected with SV40LT four days and three weeks after infection and not infected HMEC (around PD 3) and HMEC-hTERT infected with SV40LT. Only cells infected with the SV40LT-mCherry vector expressed the SV40LT antigen. **(C)** Cytometric plots of SV40 st+LT expression in SV-TO cells at p31 and p33 also demonstrated the leaking effect. In contrast, 79.4% of HMEC-hTERT infected with the SV40LT-mCherry vector at PD 6.92 and analysed by flow cytometry at PD >92.31 expressed the SV40 st+LT. The absolute percentage of SV40LT cells, where positive cells of the A488 control have been subtracted, is shown. The respective control A488 plots are the smaller ones depicted. **(D)** Representative cell cycle plots of SV-TO cells at p31 and p33 and HMEC-hTERT-SV40LT at PD >92.31. An abnormal cell cycle profile was only observed in the HMEC-hTERT-SV40LT cells where a 35.4% of cells were >4N.

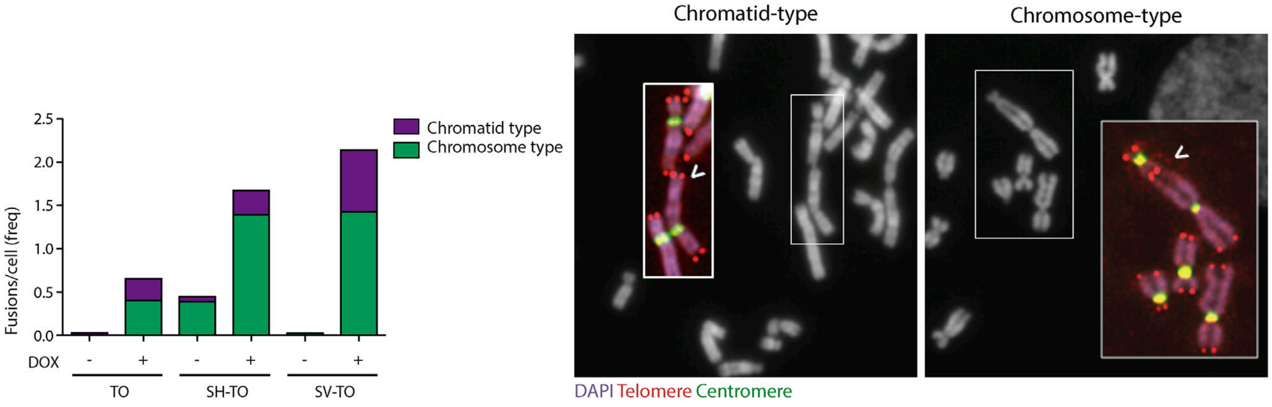

Supplementary Figure 6: $TRF2^{\Delta B\Delta M}$ expression induces chromosome and chromatid end-to-end fusions in all inducible cell lines. (A) Distribution of chromosome-type and chromatid-type fusion per cell in uninduced and induced cell lines. Most fusions were produced at G1, i.e. were of the chromosome-type, although chromatid-type fusions were also observed. **(B)** Partial images showing a chromosome-type fusion and a chromatid-type fusion. In the raw image of DAPI, the physical connexion between chromosome arms, through only one or both chromatids, is observed. The insets show the telomere and centromere status of the fusion events, confirming that each fusion event is labelled with the telomeric PNA probe.

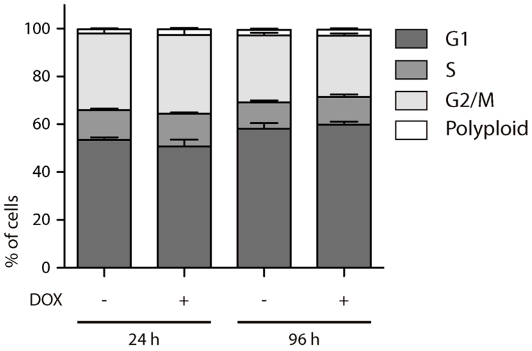

Supplementary Figure 7: Doxycycline does not alter the cell cycle profile of parental MCF-10A cells. Propidium iodide-based cell cycle analysis was performed after exposure of MCF-10A cells to 1 µg/ml DOX during 24 h or 96 h. Assay was performed in triplicate, with error bars representing + SEM. Data were analysed by Mann-Whitney U-test, comparing control *vs.* treated group, and p-values were higher than 0.05.

Supplementary Table 1: Abnormal karyotypes and chromosome end fusions in MCF-10A and inducible variants expressing $TRF2^{\Delta B\Delta M}$

Cell line	DOX treatment	Cells analysed n	% of abnormal karyotype (n)*	% of cells with fusions (n)	Fusions per cell (n)
MCF10A	-	37	10.81 (4)	2.70 (1)	0.03 (1)
TO	-	35	8.57 (3)	2.86 (1)	0.03 (1)
TO	96 h	55	45.45 (25)	36.36 (20)	0.65 (36)
SH-TO	-	36	55.56 (20)	27.78 (10)	0.44 (16)
SH-TO	96 h	46	67.39 (31)	58.70 (27)	1.67 (77)
SV-TO	-	38	71.05 (27)	2.63 (1)	0.03 (1)
SV-TO	96 h	35	82.86 (29)	65.71 (23)	2.14 (75)

* Relative to the basal karyotype (includes cells with fusions).

Supplementary Table 2: Telomere status of fusion's junction point in untreated or DOX-treated cell lines

Cell line	DOX treatment	Cells analysed n	Total fusions n	% of telomere negative fusions (n)	% of telomere positive fusions (n)
TO	-	35	1	100.00 (1)	0 (0.0) (0)
TO	96 h	55	35	5.71 (2)	94.29 (33)
SH-TO	-	36	14	78.57 (11)	21.43 (3)
SH-TO	96 h	46	72	15.28 (11)	84.72 (61)
SV-TO	-	38	1	100.00 (1)	0.00 (0)
SV-TO	96 h	35	71	8.45 (6)	91.55 (65)

[*] The number of fusion events where the telomere status of the junction point was resolved is less than the total number of fusions due to hybridization technique pitfalls.

Supplementary Table 3: Anaphase cells containing chromatin bridges after TRF2$^{\Delta B\Delta M}$ expression

Cell line	DOX treatment	Cells examined (n)	% of cells with anaphase bridges (n)
TO	-	215	3.72 (8)
TO	96 h	318	45.60 (145)
SH-TO	-	243	18.93 (46)
SH-TO	96 h	490	53.06 (260)
SV-TO	-	736	4.21 (31)
SV-TO	96 h	608	38.16 (232)

Supplementary Table 4: Numerical chromosome changes after persistent or transient telomere deprotection

Cell line	DOX treatment	Cells examined (n)	2N		4N	
			% of euploid (n)	% of aneuploid (n)	% of euploid (n)	% of aneuploid (n)
TO	-	439	89.98 (395)	9.79 (43)	0.23 (1)	0.00 (0)
TO	96 h	285	83.86 (239)	14.39 (41)	1.40 (4)	0.35 (1)
TO	5x24 h	451	94.24 (425)	4.88 (22)	0.67 (3)	0.22 (1)
SH-TO	-	539	80.52 (434)	14.10 (76)	2.60 (14)	2.78 (15)
SH-TO	96 h	284	75.70 (215)	13.73 (39)	3.52 (10)	7.04 (20)
SH-TO	5x24 h	452	88.50 (400)	8.19 (37)	1.33 (6)	1.99 (9)
SV-TO	-	580	93.28 (541)	4.66 (27)	1.21 (7)	0.86 (5)
SV-TO	96 h	419	90.45 (379)	6.44 (27)	1.19 (5)	1.91 (8)
